# The rise of Parkinson’s disease is a global challenge, but efforts to tackle this must begin at a national level: a protocol for national digital screening and “eat, move, sleep” lifestyle interventions to prevent or slow the rise of non-communicable diseases in Thailand

**DOI:** 10.3389/fneur.2024.1386608

**Published:** 2024-05-13

**Authors:** Roongroj Bhidayasiri, Jirada Sringean, Saisamorn Phumphid, Chanawat Anan, Chusak Thanawattano, Suwijak Deoisres, Pattamon Panyakaew, Onanong Phokaewvarangkul, Suppata Maytharakcheep, Vijittra Buranasrikul, Tittaya Prasertpan, Rotjana Khontong, Priya Jagota, Araya Chaisongkram, Worawit Jankate, Jeeranun Meesri, Araya Chantadunga, Piyaporn Rattanajun, Phantakarn Sutaphan, Weerachai Jitpugdee, Marisa Chokpatcharavate, Yingyos Avihingsanon, Chanchai Sittipunt, Werasit Sittitrai, Grisada Boonrach, Aekamorn Phonsrithong, Pichit Suvanprakorn, Janprapa Vichitcholchai, Tej Bunnag

**Affiliations:** ^1^Chulalongkorn Centre of Excellence for Parkinson’s Disease and Related Disorders, Department of Medicine, Faculty of Medicine, Chulalongkorn University and King Chulalongkorn Memorial Hospital, Thai Red Cross Society, Bangkok, Thailand; ^2^The Academy of Science, The Royal Society of Thailand, Bangkok, Thailand; ^3^National Electronics and Computer Technology Centre, Pathum Thani, Thailand; ^4^Sawanpracharak Hospital, Nakhon Sawan, Thailand; ^5^Department of Rehabilitation Medicine, King Chulalongkorn Memorial Hospital, Thai Red Cross Society, Bangkok, Thailand; ^6^Chulalongkorn Parkinson's Disease Support Group, Department of Medicine, Faculty of Medicine, Chulalongkorn Centre of Excellence for Parkinson's Disease and Related Disorders, Chulalongkorn University and King Chulalongkorn Memorial Hospital, Bangkok, Thailand; ^7^Faculty of Medicine, Chulalongkorn University, Bangkok, Thailand; ^8^Thai Red Cross Society, Bangkok, Thailand

**Keywords:** Parkinson’s disease, Thailand, digital interventions, non-communicable diseases, prevention, lifestyle interventions

## Abstract

The rising prevalence of Parkinson’s disease (PD) globally presents a significant public health challenge for national healthcare systems, particularly in low-to-middle income countries, such as Thailand, which may have insufficient resources to meet these escalating healthcare needs. There are also many undiagnosed cases of early-stage PD, a period when therapeutic interventions would have the most value and least cost. The traditional “passive” approach, whereby clinicians wait for patients with symptomatic PD to seek treatment, is inadequate. Proactive, early identification of PD will allow timely therapeutic interventions, and digital health technologies can be scaled up in the identification and early diagnosis of cases. The Parkinson’s disease risk survey (TCTR20231025005) aims to evaluate a digital population screening platform to identify undiagnosed PD cases in the Thai population. Recognizing the long prodromal phase of PD, the target demographic for screening is people aged ≥ 40 years, approximately 20 years before the usual emergence of motor symptoms. Thailand has a highly rated healthcare system with an established universal healthcare program for citizens, making it ideal for deploying a national screening program using digital technology. Designed by a multidisciplinary group of PD experts, the digital platform comprises a 20-item questionnaire about PD symptoms along with objective tests of eight digital markers: voice vowel, voice sentences, resting and postural tremor, alternate finger tapping, a “pinch-to-size” test, gait and balance, with performance recorded using a mobile application and smartphone’s sensors. Machine learning tools use the collected data to identify subjects at risk of developing, or with early signs of, PD. This article describes the selection and validation of questionnaire items and digital markers, with results showing the chosen parameters and data analysis methods to be robust, reliable, and reproducible. This digital platform could serve as a model for similar screening strategies for other non-communicable diseases in Thailand.

## 1 Introduction

### 1.1 The global rise in Parkinson’s disease

Parkinson’s disease (PD) is reported to be the fastest-growing neurological disorder worldwide in terms of mortality, disability and age-standardized prevalence (1, 2). Data from the World Health Organization (WHO) in 2019 showed that globally, the number of individuals with PD had reached over 8.5 million (3), a substantial rise from the 6.1 million cases reported in 2016 and the 2.5 million in 1990 (4). The projected global burden of PD is likely to be over 17 million cases by 2040 (2). This particularly steep rise in PD incidence and prevalence seen over the last few decades compared to previous years has led to some describing the phenomenon as having characteristics of a “pandemic,” a term normally reserved for infectious diseases (2). However, the overall picture is complex, and this description has been challenged since it focuses primarily on exogenous drivers of disease causality and does not take into account factors such as the increase in PD duration in recent decades, the comparative decline in other neurological diseases, and the limitations of a wide range of data sources used to ascertain these prevalence estimates (5).

In terms of what might be responsible for the rise in cases, it is important to recognize that PD is a complex, multisystem disorder, and evidence suggests there are multiple, and most likely overlapping, drivers and risk factors that could potentially contribute to this trend. One key factor is likely to be the changing population demographics globally with an increase in the proportion of elderly people due to improvements in health and life expectancy. Aside from demographic drivers, there is also evidence that genetics and environmental factors, such as a reduction in smoking rates and an increase in exposure to pollutants and pesticides, also contribute to the rise in PD (2, 6–11). Ethnic differences have also been suggested to increase PD risk, although these differences might be explained by disparities in access to healthcare across countries and regions (7). There is also preliminary evidence that associations between some environmental and lifestyle factors and PD may be modified by the person’s genotype (9). Therefore, in our efforts to control the rise of PD, a critical aspect to consider is minimizing what in many cases are modifiable risk factors with strategies that can include simple lifestyle changes (12, 13). Similarly, we need to be aware of independent protective effects, namely consumption of coffee, nicotine, green and black tea, urate, non-steroidal anti-inflammatory drugs, and physical activity that have been identified in various studies (6, 14).

Although the rise in PD prevalence is a global burden, the reported PD prevalence patterns are not uniform across all world regions. Particularly steep increases have been observed in highly populous nations in Asia (15), and in low-to-middle income countries (LMICs) (12, 16, 17). Using data from the Global Burden of Disease (GBD) 2019 study, it has been estimated that the prevalence of PD over the period from 1990 to 2019 increased by 256% in East Asia, 216% in South Asia, 174% in South-East Asia, and 57% in Central Asia (18). This pattern of rising PD prevalence is likely to be similar in Thailand which has an increasingly aging population (19.46% are over 60 years of age) (19). However, other contributing factors to the increasing number of cases are likely to be increased awareness of PD due to various educational campaigns, better therapeutic options, and increased availability and accessibility to treatment within Thailand (16).

In the face of this considerable and escalating healthcare burden, the traditional “passive” approach to PD management (waiting for patients to seek treatment) is unlikely to meet the needs of an expanding PD population and it is likely that, as a result, the quality of care for patients with PD in Thailand will decline due to insufficient manpower and resources. Alternative approaches that focus on prevention and early detection of PD are needed to minimize the treatment burden on the healthcare system.

### 1.2 Healthcare and societal implications of undiagnosed or untreated Parkinson’s disease

When the diagnosis of PD is delayed and it is allowed to progress to a more advanced stage before appropriate interventions are put in place, there are substantial negative consequences for PD patients themselves, their families and carers, and for wider society.

Firstly, if a late diagnosis is made, the patient will inevitably be exhibiting significant motor and nonmotor symptoms that require effective management, so the direct cost of treatment are likely to be more expensive than for a patient diagnosed early care (20, 21). Secondly, the patient is more likely to experience complications and/or adverse events from the disease itself and from the treatment required to control their symptoms due to the progression of their disease, and this will impact their quality of life (QoL). A national survey of over 600 people with PD (Hoehn and Yahr stage II–IV) in the USA revealed a high prevalence of uncontrolled motor and nonmotor symptoms (86% of respondents reported at least 1% and 10% reported ≥ 10 symptoms) and critical events, such as hospitalization or frequent falls, despite medical treatment (22). This high symptom burden was found to have a substantial negative impact on the individual’s QoL, with uncontrolled symptoms being most associated with poor QoL. Thirdly, advanced and late-stage PD patients require substantially more supportive and personal care and consume more healthcare resources, increasing the burden on carers, families, and society as a whole (23, 24). The overall cost of treatment is known to be significantly more expensive for advanced compared with early-stage PD. Although there are no specific cost effectiveness studies relating to PD management in Thailand, studies from the US and Europe have found that advanced PD is associated with significant societal costs, both direct and indirect, as they become increasingly debilitated and require more complex care (20, 21). One important societal impact of PD progression is the loss of a productive workforce as patients with late-stage PD are unlikely to be able to continue working. The healthcare burden of PD also extends to families and caregivers in terms of a negative impact on their own health and mental wellbeing but also can have economic implications as they may be less able to undertaken work due to caring for a person with PD (25, 26).

Achieving timely and effective diagnosis of PD is heavily reliant on national economics and healthcare infrastructure in order to provide the necessary resources to meet these demands (12). Thailand has a total population of almost 69 million and is served by 927 government hospitals, 363 private hospitals, and 9,768 primary health care units with 99.5% of the population having health protection coverage (27). However, with a large proportion of the population living in poorer rural areas of the country, previous experience has identified considerable challenges in terms of healthcare delivery to such a diverse population, particularly where resources and access to specialists expertise may be limited (28).

### 1.3 Diagnosing early-stage Parkinson’s disease

Recognizing the high cost of PD treatment as the disease progresses, a key management strategy needs to be the early identification of PD cases. An accurate clinical diagnosis of PD at the earliest possible stage is essential to timely and effective management and optimal long-term outcomes for patients (29). Among non-specialist clinicians, however, low levels of diagnostic accuracy have been reported ranging from 65% to 93%, especially when evaluating early-stage PD (30, 31). A recent systematic literature review highlighted that diagnostic accuracy of PD among general practitioners in the primary care setting is hard to ascertain as there are no well-conducted studies. However, the studies identified in the review did reveal some specific problems, such as knowledge gaps about PD and delays in referral due to lack of timely symptom identification (32). Accurate diagnosis is therefore best achieved by consultation with movement disorder neurologists; however, they are not always easily accessible, especially in LMICs, leading to delays in obtaining a definitive diagnosis and appropriate treatment (16). Misdiagnosis of PD for other types of movement disorder or other medical conditions is also relatively common among patients with early-stage disease, with rates as high as 20%–30% reported (31, 33). This is particularly evident for cases of early-onset PD, a term which describes disease occurring in people over the age of 21 but younger than 50 years of age (34) and which now accounts for 8%–10% of the PD population. However, this figure may vary across different ethnic groups due to variations in underlying genetics. Non-specialist clinicians may not be expecting what is generally perceived as a condition of the elderly, in these younger subjects. Added to this, in certain world regions, including some countries in Africa and parts of Asia, PD is associated with supernatural beliefs and sufferers may face extreme marginalization, stigmatization and persecution for having the disease, resulting in social isolation, poor attendance at healthcare facilities, lack of access to care, definitive diagnosis, and treatment (35). In the experience of the largest tertiary for PD management in Thailand, the Chulalongkorn Centre of Excellence for Parkinson’s Disease and Related Disorders (ChulaPD, www.chulapd.org) in Bangkok, PD patients are generally diagnosed when they have already had symptoms on average for 3–5 years, so they are often very symptomatic (12). Considering that dopaminergic neuron degeneration is already severe in the early stage of PD, reaching at least 60%, achieving a definitive diagnosis after several years of being symptomatic is likely to be too late. While these findings cannot be generalized to all centers in the country who see PD patients, it is possible that in less specialized institutions the situation may be even worse.

The significant problem of the rise in the PD population globally is, however, only the tip of the iceberg, and the many undiagnosed cases in the early disease stage—a time when therapeutic interventions would have most value—need to be considered in healthcare resource planning. The rise in PD is recognized as a global challenge, but efforts to tackle it need to begin at a national level (36). Effective diagnostic strategies need to be put in place but in many countries, including Thailand and other LMICs, there is a lack of specialist movement disorders neurologists and limited healthcare resources to achieve this (12). In Thailand, up to 80% of PD patients have never been assessed by a specialist neurologist, only by non-specialist clinicians or not being evaluated by medical practitioners at all (37). There are also a limited number of specialist neurologists nationally who are skilled in diagnosing and treating patients with movement disorders such as PD, and over half of those are located in urban areas (37, 38).

As will be discussed in detail later, it is in this context that digital health technologies can have an important role to play, especially in LMICs. They offer a practical solution to help clinicians, particularly non-specialists, and other healthcare professionals involved in PD patient care with a supplementary screening tool to help identify patients in the earlier stages of PD. The cost-effectiveness of early detection with such digital solutions and whether the health gains they can provide justify the cost of investment has been of considerable interest. A study undertaken in the USA evaluated the cost-effectiveness of sensor-based interventions for PD in terms of quality-adjusted life years and associated costs. The results indicated that the digital interventions are cost-effective in this setting, providing both health gains and economic benefits, particularly when deployed for community health screening in public places such as health fairs and pharmacies (39). This approach is now a priority in Thailand and may be particularly useful for targeting diverse populations across both urban and rural regions of the country (36).

### 1.4 The need for Parkinson’s disease prevention strategies

Up until recently, the management of neurodegenerative diseases has focused primarily on diagnosis and treatment, and “prevention” has not been perceived as a valid target since for many years these diseases have been considered unpreventable. However, in the light of accumulating evidence, there is now an urgent call from the World Health Organization (WHO) and several other professional organizations to include prevention as part of the overall brain health strategies (17, 40–42). In the case of PD, this change in focus has arisen as a result of the accumulating research evidence demonstrating the complex interplay of causative and risk factors that underpin the pathology of PD. PD, which was previously thought of as a localized brain disease, is now recognized as a multisystem disorder involving other organ systems with both genetic and environmental factors contributing to its development (43, 44). This wider knowledge has highlighted the range of possibilities for intervention, making prevention of PD a real possibility and something that should be considered as a priority. Similarly, in other neurodegenerative conditions, such as Alzheimer’s disease, there is an increasing focus on prevention trials and the inclusion of subjects at earlier, prodromal stages as targets for early intervention (45). A focus on prevention of PD rather than just management is expected to help reduce the numbers of PD cases moving forward, as has been done for other medical conditions such as cardiovascular disease and some types of cancer (2, 7, 17). In recognition of this, the WHO and others have stressed the need for urgent action, not only to raise awareness of the current and future healthcare challenges of an expanding PD population, but also to focus on developing preventative, or neuroprotective strategies that can be applied at a population level before symptoms develop.

PD is known to have a long prodromal phase which can extend up to 20 years prior to clinical diagnosis (46–48). This is the latent period over which there is a gradual development of PD pathology prior to the emergence of the characteristic and observable motor signs and symptoms that enable a clinical diagnosis, and which offers a window of opportunity for disease prevention (47, 49). Currently, no definite disease-modifying therapies have been identified for PD that can slow, stop or reverse the progression of disease, although considerable research in this field is ongoing (50, 51), so clinical management relies on symptomatic treatment. Even if an effective disease-modifying therapy was identified tomorrow, due to the prolonged time needed for clinical trials and regulatory approval, it might be a decade or more before it could be used in the clinical practice setting (52). In addition, both the WHO and the European Association for Neurology (EAN) recognize the significant burden associated with the treatment of established PD, and have highlighted the importance of prioritizing exploration of effective prevention strategies, currently one of the top six global research gap categories in neurology (17, 40, 42). The WHO has recently issued a position paper outlining its commitment to brain health across each person’s lifetime in order to optimize overall population health and wellbeing, and this includes a focus on healthy behaviors (41).

Disease “prevention” in the field of public health, however, is not a single concept, but can be classified as primary, secondary, and tertiary, based on the timing and target of the intervention (7). Primary prevention aims to identify and remove the cause of the disease so that it never develops; secondary prevention tackles the disease processes at an early stage and intervenes to prevent or slow symptoms from developing, while tertiary prevention describes efforts to slow progression and minimize the burden of established disease. The importance and interest in PD prevention was highlighted by an international conference held in 2021 entitled “Planning for Prevention of Parkinson: A Trial Design Symposium and Workshop.” This focused specifically on the challenges of design and implementation of prevention trials in PD, in particular, who should be recruited, what interventions should be tested, and what endpoints should be measured in order to provide meaningful and robust outcomes data (53–56).

### 1.5 Screening for prodromal and early Parkinson’s disease

**Figure 1:**
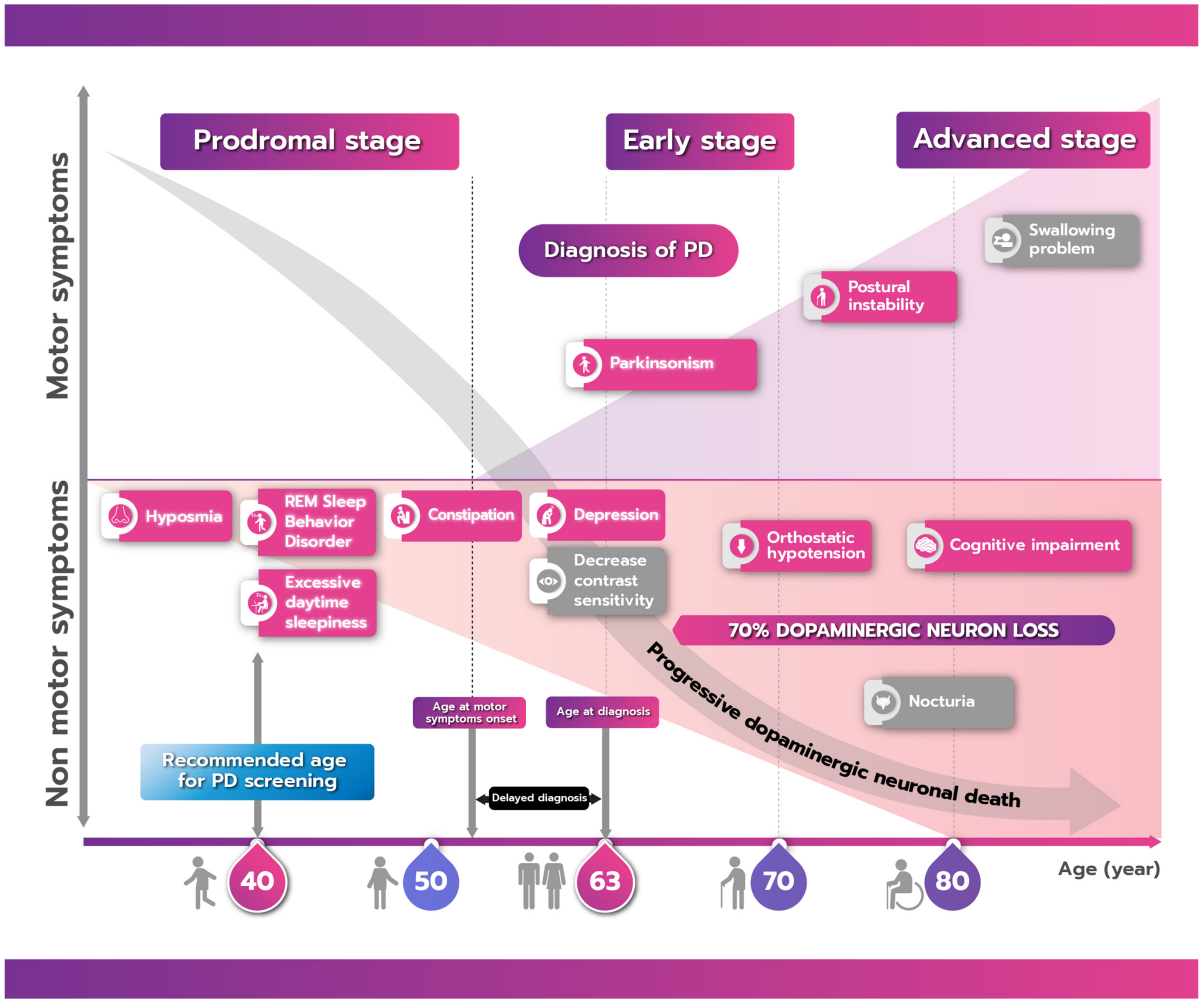
The natural history of Parkinson’s disease (PD) showing the progression of motor and nonmotor symptoms from the prodromal to advanced stage. There is commonly a delay between onset of motor symptoms and a confirmed clinical diagnosis of PD. Boxes shaded in red/pink are clinical parameters that can be screened either by using a questionnaire or digital application while those in gray cannot. Due to the long prodromal period of PD, the recommended age for screening is 40 years.

PD has a long natural history, with evidence of neurodegeneration becoming evident 10–20 years before the onset of motor symptoms (47). Data from the Thailand Parkinson’s Disease Registry shows that the average age of PD patients at diagnosis is approximately 63 years of age (57). It is logical, therefore, that in the timeline of PD development and progression, any preventive strategies should be implemented before the onset of degeneration (Figure 1). Therefore, the target demographic for a national screening program in Thailand to determine PD risk are people from the age of 40 years onwards, approximately 20 years before the usual emergence of PD motor symptoms. This timing is supported by evidence from clinical, pathological, and neuroimaging studies, which show that non-motor symptoms are observable 10–20 years before motor symptoms are evident, with dopaminergic neurodegeneration in the substantia nigra of the brain observed up to 6 years before diagnosis and other neuropathology likely occurring prior to this in other parts of the central nervous system (46, 58).

In order to determine who might benefit from possible PD prevention strategies, suitable candidates first need to be identified based on validated markers. Evidence now suggests that there are a range of motor and nonmotor prodromal markers, both clinical [for example, rapid eye movement (REM) sleep behavior disorder (RBD), constipation, or olfactory changes] and pathological (neuronal loss and Lewy body pathology), which are associated with the subsequent development of PD (59–61). Of all prodromal symptoms, RBD has the highest predictive value for the future development of PD (60, 62). Investigation of the evolution of prodromal markers in a cohort of patients with idiopathic RBD showed varying times of onset before a definitive diagnosis of PD (46). Among the nonmotor symptoms, olfactory loss was first to develop, often occurring >20 years before confirmed PD, impaired color vision, constipation, and erectile dysfunction, occurring 10–16 years before, with urinary dysfunction and cognitive decline being detected 7–9 years before. Motor impairments, such as ability on the alternate tap test, could be observed up to 8 years prior to diagnosis. A case-controlled study using data from the Health Improvement Network primary care database in the UK of 8,166 individuals with and 46,755 individuals without PD over the period from 1996 to 2012 also confirmed a range of prediagnostic features that could be detected several years before a clinical diagnosis of PD (63). An algorithm for risk of diagnosis of PD within 5 years was calculated using multivariate logistic regression. The factors that were found to be independently and significantly associated with a subsequent diagnosis of PD were tremor, constipation, depression or anxiety, fatigue, dizziness, urinary dysfunction, balance problems, memory problems and cognitive decline, hypotension, rigidity, and hypersalivation (64).

Interest in prodromal PD and the number of identified markers have risen considerably in the past decade (60). In 2015, recognizing this growing interest, the International Parkinson and Movement Disorder Society (MDS) published a set of criteria for prodromal PD to be used in the research setting to help standardize identification and diagnosis (59, 62). These are being regularly updated as new evidence becomes available. The prodromal markers identified offer a promising tool to help determine likely conversion to PD but further longitudinal studies in a wider range of PD cohorts are needed to confirm and enhance these findings and improve diagnostic accuracy (59, 65, 66). An MDS Web Portal is available that aims to encourage prodromal PD research and information exchange and provides a practical calculator of prodromal PD probabilities using the most recent criteria (59).

In the “real world” setting, population screening to enable prompt recognition of these important prodromal PD markers offers an opportunity to better understand disease progression and for early intervention with possible preventative strategies (or timely treatment if established disease recognized). Various screening initiatives have been evaluated for use in clinical practice settings in different countries to identify people at risk of developing PD. These have focused on early motor and nonmotor PD symptoms as well as prodromal markers and generally used survey-based methodology with patient-completed questionnaires, in some cases combined with objective testing. RBD, the strongest marker of prodromal PD, has been evaluated as a screening tool in a Canadian community-based study (67). This used a multistep approach using RBD screening questionnaires, and those who passed both screening stages (29 patients) underwent confirmatory polysomnography. This screening strategy was found to have a positive predictive value of 66, and 63% of those who underwent full polysomnography met the criteria for prodromal PD. The PREDICT-PD study undertaken in the UK was the first to systematically combine risk factors for PD in a general population cohort (68, 69). The study included participants aged 60–80 years without PD who completed an online survey that incorporated validated questionnaires on depression, RBD, and early nonmotor features of PD. They also underwent genetic testing, smell tests, and an evaluation of finger-tapping speed. The results of the study, and its longitudinal follow-up, enabled development of an online algorithm that provides a simple tool to help prospectively identify PD risk.

### 1.6 The value of digital technologies for Parkinson’s disease assessment

Classical objective tests for the assessment of PD include neuroimaging or analysis of blood or cerebrospinal fluid (CSF) (70–72), but these are generally invasive and can be costly. In addition, they may not be accessible in every setting or may require specialist knowledge to undertake. Portable, digital methods for assessment offer a valuable alternative that can potentially improve delivery of, and access to, clinical care. Importantly, digital technologies may be of particular value in LMICs to improve healthcare delivery where human and financial resources are limited (73). While there may be challenges associated with the introduction of these new technologies into existing workflows and healthcare systems in LMICs (74), there is now considerable evidence of their feasibility and utility in clinical use, in particular allowing remote assessment and collection of data (75, 76). Mobile phones are now widely used in most countries around the world which allows easier connectivity between citizens and healthcare providers, particularly in remote rural areas (77). In fact, one of the key learnings from the COVID-19 pandemic, particularly for LMICs, was that embracing digital technologies has the potential to not only improve the ability to respond to public health challenges but can also strengthen primary healthcare systems delivering care (78).

Digital tools and other innovative technologies are already widely used in the fields of neurology and movement disorders to assist clinicians with diagnosis, monitoring and treatment decisions, and continue to be the focus of considerable research and development (12, 79–82). Smartphone applications and smartwatches, along with machine learning technology, are being increasingly used to collect activity data that can aid the diagnosis and monitoring of PD (83–87). They have also been evaluated for use in gathering outcomes data in clinical trials (88). Smartphones are relatively easy to use, familiar to most people, relatively low cost, non-invasive and allow data collection by non-specialists, either in-person or remotely. The various sensors that are typically incorporated in smartphones, such as accelerometers, gyroscopes, microphones, make them ideally suited for gathering information from people with PD. Testing activities for PD diagnosis using smartphone applications have been used successfully and with good accuracy for analysis of gait (89, 90), spoken voice (91, 92) and tremor (93, 94). Finger movement while typing has also shown promising results with high accuracy for the early detection of PD (95, 96). Data gathered from these activities by the smartphone’s sensors are processed for extraction of diagnostic features, which can include general statistical analyses (mean, standard deviation, percentiles), but also measurements of time and frequency. The extracted features are then fed into a machine learning classifier, such as neural networks (NN), decision tree, and support vector machine (SVM), to distinguish between PD and non-PD cases (90, 92, 97). Mobile technology and the use of digital markers has recently been successfully validated for the self-administered, at-home assessment of Alzheimer’s disease risk to allow remote screening to support clinical assessment (98). Another digital strategy that is currently being evaluated in a clinical trial in people with Alzheimer’s disease (NCT05027789), is what is termed a “Brain Boosters” intervention, which combines memory training and promotion of healthy lifestyle behaviors which are implemented and monitored using a digital application (99).

The research team at ChulaPD has extensive experience in the field of digital technologies, with a track record of publication on device/sensor development and applications (96, 100–104). To date, the devices and technologies that have been developed have been focused on strategies for patients who have already been diagnosed with PD. They have been employed to improve the accuracy of PD diagnosis and monitoring and to help inform suitable therapeutic interventions, as well as being used as assistive technologies to aid intractable symptoms such as freezing of gait using a laser cane (105), intractable tremor using the specially-designed “Tremor’s Glove” (106), and for assessment of nocturnal hypokinesia with nocturnal devices.

Recognizing the importance of early detection of PD, the team has applied their digital expertise to the Parkinson’s disease risk survey in order to develop screening strategies for the Thai population that can detect subtle symptoms and undertake risk evaluation. They have developed a smartphone application that collects tremor, gait, finger tapping and voice data to detect early signs of PD and incorporates newly developed speech tests using Thai sentences and sustained vowels, along with a finger pinching test. The process for selection and validation of these digital markers is described in Methods, section 2.3.

Thailand is an example of a small LMIC that has benefitted from considerable social and economic development in recent decades, and has an established program of universal healthcare in place for its citizens (12). These attributes make it well placed for implementation of a national screening program using digital technology, as it proposed for the Parkinson’s disease risk survey. In addition, due to national distribution by the Chula PD team of the laser cane device that helps identify moderate-to-advanced stage PD patients with gait problems, there is already an existing clinical network that can be used to assist in identification of patients in the early stage of PD.

### 1.7 The Thai Parkinson’s disease risk survey

**Figure 2:**
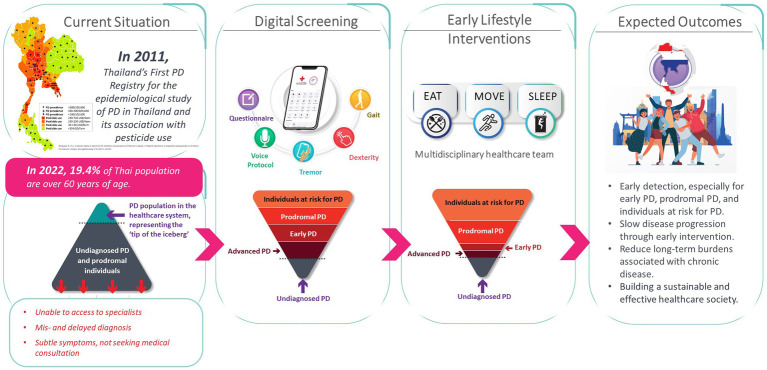
Rationale and objectives of the Parkinson’s disease risk survey screening program.

The Parkinson’s disease risk survey (Thai Clinical Trials Registry identification number: TCTR20231025005) is being undertaken to evaluate a digital population screening platform that will allow identification of undiagnosed cases of PD, which may include early and more advanced cases, within the Thai population aged over 40 years. It is also hoped that the study will identify those in the prodromal stage as well as at-risk individuals who can be targeted with preventive lifestyle interventions. The overall rationale and objectives of the national digital screening program and its expected outcomes are outlined in Figure 2.

**Figure 3:**
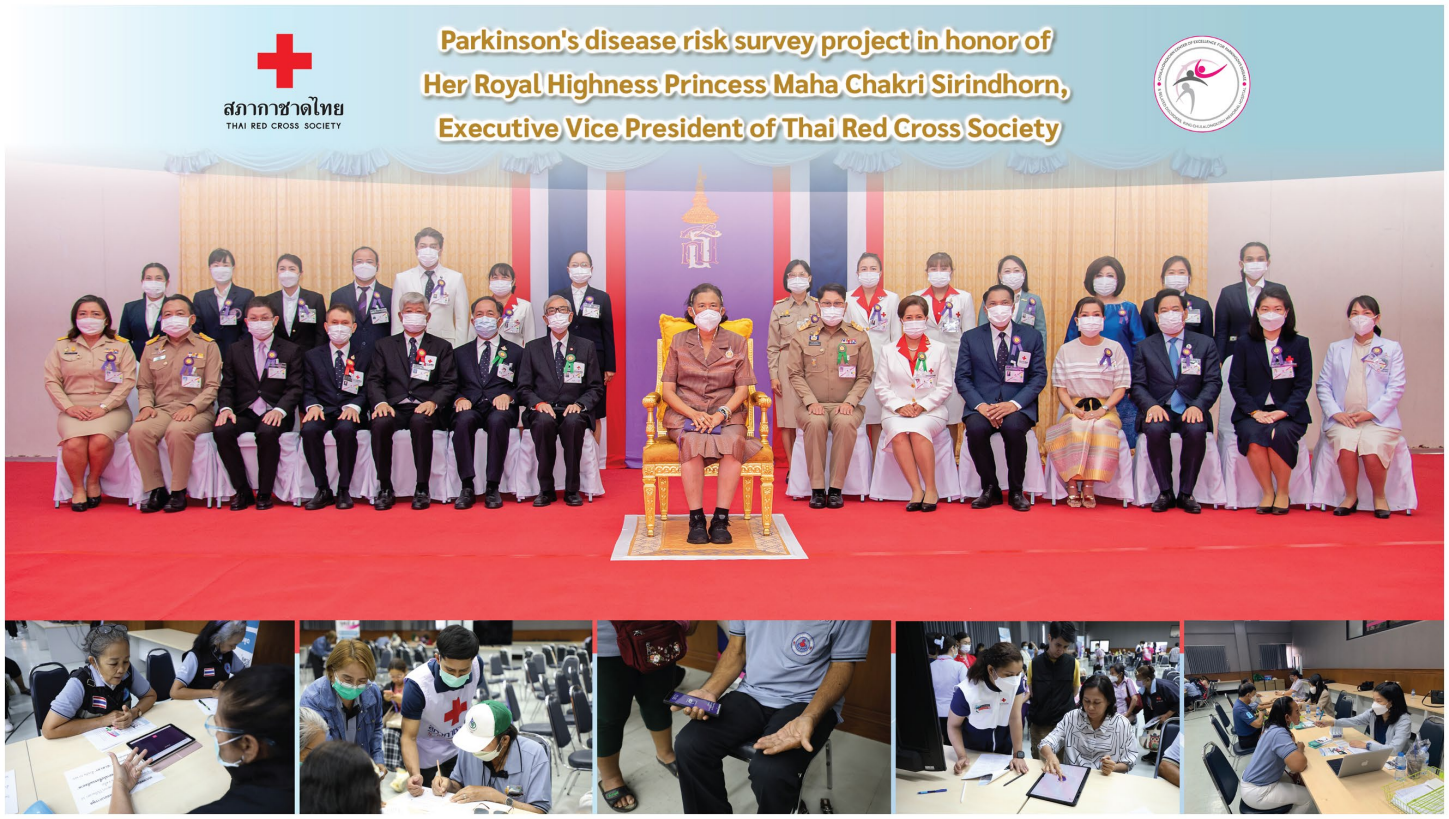
The Parkinson’s disease risk survey project implemented in honor of Her Royal Highness Princess Maha Chakri Sirindhorn, Executive Vice President of Thai Red Cross Society. Top: Her Royal Highness and the multidisciplinary team involved in the study’s design, validation, and implementation at the official opening event; bottom: subjects being screened with the digital tools.

The Parkinson’s disease risk survey project has been implemented in collaboration with the Thai Red Cross Society and is celebrated under the auspicious occasion of Her Royal Highness Princess Maha Chakri Sirindhorn, Executive Vice President of the Thai Red Cross Society. The design of the study involved collaboration between a multidisciplinary group of experts in the field of PD, comprising movement disorders neurologists, led by RB, project manager, general neurologists, internists, data science engineers with experience in machine learning, PD nurse specialists, patient representatives, a physical therapist, a dietitian, and policymakers. The campaign was officially opened on the 18th of January 2024 by the Princess on the occasion of the implementation of the first field study in Nakhon Sawan Province, located in the north-central part of Thailand (Figure 3).

Prodromal PD refers to the stage at which individuals do not fulfill diagnostic criteria for PD (i.e., bradykinesia and at least one other motor sign) but do exhibit signs and symptoms that indicate a higher-than-average risk of developing motor symptoms and a diagnosis of PD in the future (62). The digital platform will be deployed nationally and allow the collection of data on the person’s speech, hand tremor, finger dexterity, and mobility, which will be analyzed alongside information obtained from a questionnaire about PD symptoms completed by the participants. The outcomes will enable a range of lifestyle interventions to be implemented in those determined to be at risk of developing PD in the future or suitable treatment for those considered to have early PD.

Evidence is emerging that interventions such as regular exercise, consuming a more Mediterranean-style diet, and ensuring adequate sleep quality can potentially help slow disease progression and the transition from the prodromal stage to clinical PD. Once subjects who may have prodromal have been identified through national screening (Phase 1 of the project), these lifestyle interventions—namely EAT, MOVE and SLEEP—can be implemented subsequently on a personalized basis with the objective of preventing or minimizing the development of PD within Thailand (Phase 2 of the project) (12). It is anticipated that this digital platform could also act as a model for similar screening strategies for other non-communicable diseases in Thailand.

This manuscript describes the overall design and implementation of Phase 1 of the Parkinson’s Disease Risk Survey study in Thailand, including the development and validation of the questionnaire and markers that comprise the digital platform, and the procedure for extraction and analysis of the data using machine learning tools that will identify subjects at risk of developing, or with early signs of PD. The study is currently in the early stages of implementation based on this validated methodology, and participants’ data are being collected and analyzed on an ongoing basis, and will be reported separately in due course.

## 2 Methods and analysis

### 2.1 Overall study design

The Parkinson’s disease risk survey aims to target all Thai citizens above 40 years of age. The survey will be implemented in three ways: (1) individual citizens can download the app and complete the questionnaire and tests themselves, (2) field studies will be undertaken by the ChulaPD research team across different provinces in collaboration with provincial public health offices, provincial hospitals, provincial Red Cross chapters, and district Red Cross branches, and (3) digital screening stations will be set up in every provincial hospital across Thailand. The composition of the assessment package that comprises the digital platform is shown in Table 1.

**Table 1:** Composition of the digital assessment package for national Parkinson’s disease screening in Thailand.

Test	Description	Program picture	
Questionnaire (yes or no)	10 motor symptom questions 5 nonmotor and prodromal symptom questions 5 atypical parkinsonism/red flag symptom questions	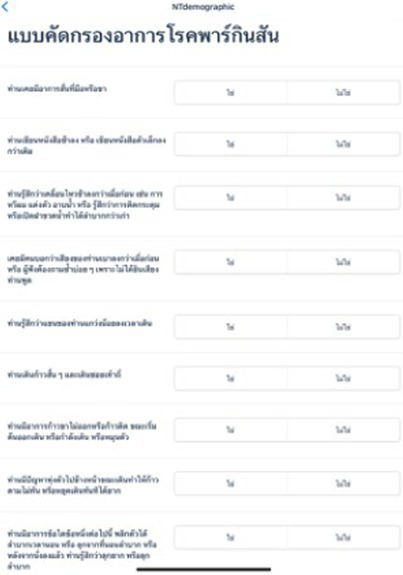	
**Voice analysis**			
Phonation	Ask participant to say the word “Ah” loudly for 10 s.	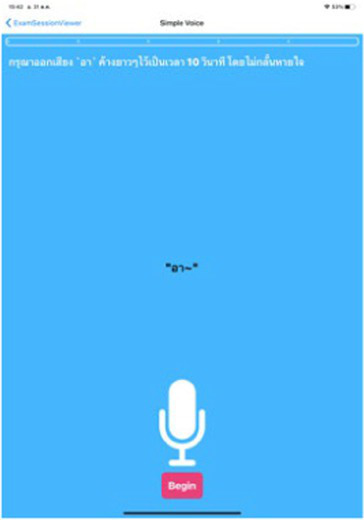	
Prosody and articulation	Ask participant to say the sentence “ยายพาหลานไปซื้อขนมที่ตลาด.”	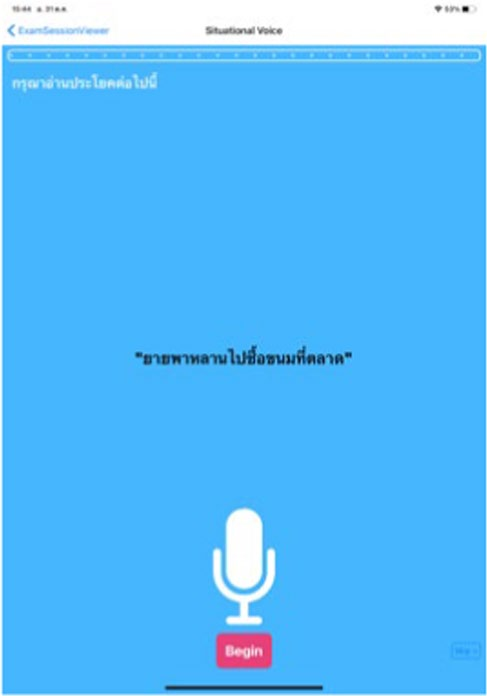	
**Motor analysis**			
Tremor tests	Rest tremor (put the device on the participant’s hand while it is resting on their lap for 20 s) Postural tremor (the participant holds the device in their hand with their arm outstretched against gravity for 20 s) *Measure the tremor of both hands but one hand at the time*	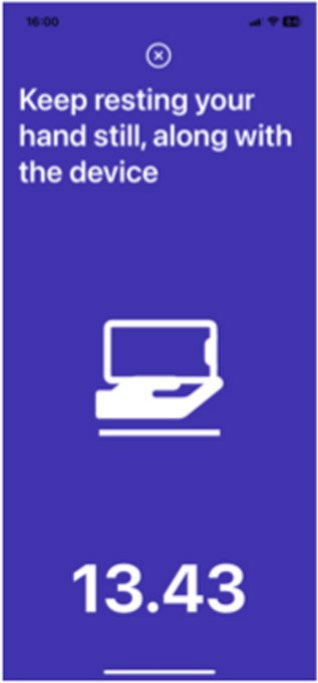 Rest tremor	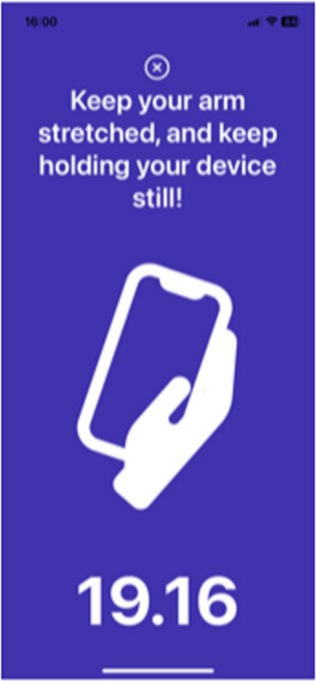 Postural tremor
Alternate finger tapping	Ask the participant to alternately tap with the index and middle fingers as fast as possible for 10 s. *Test both hands but one hand at the time*	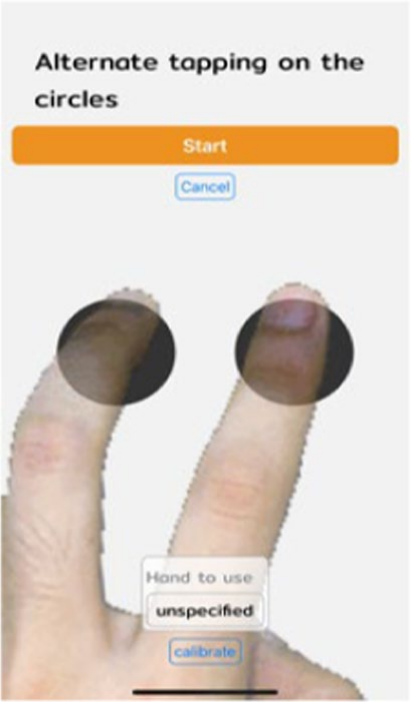	
Pinch-to-size test	Ask the participant to pinch the finger to match the different sizes of the circle shown on the screen 5 times. *Only test the dominant hand*	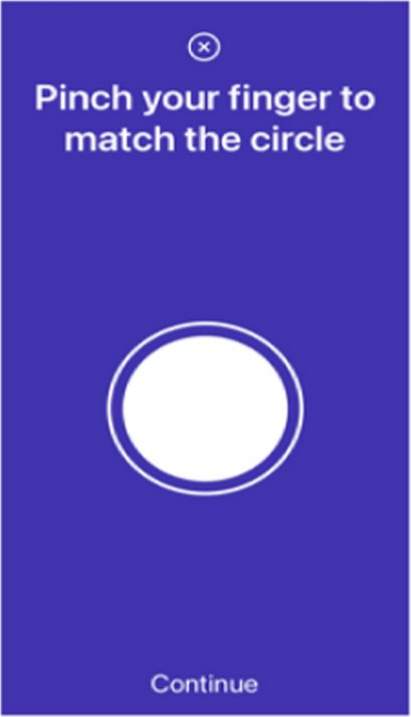	
Gait test	Ask the participant to put the device in their pocket and walk straight for 3 meters, turn around and then walk back.	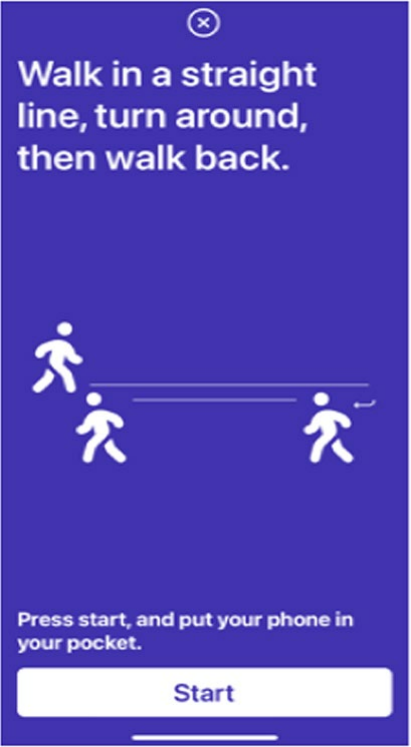	
Balance test	Ask the participant to put the device in their pocket and stay completely still for 15 s.	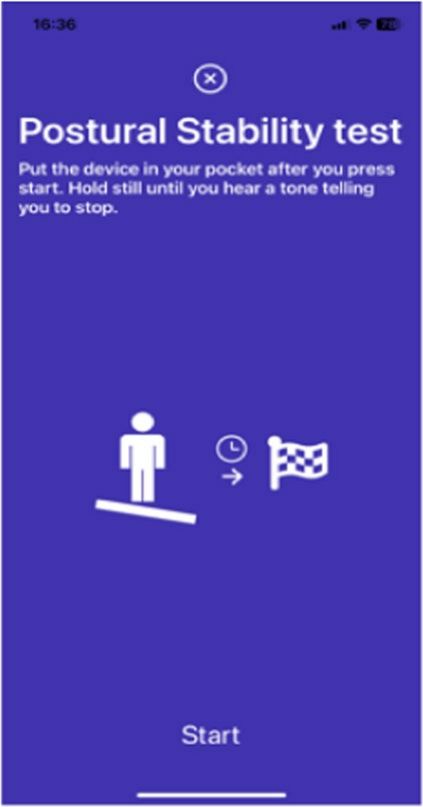	

Subjects who participate in the national screening study will be asked to give written informed consent before the enrolment in accordance with the declaration of Helsinki (IRB No.0804/65). They will be asked to complete a validated 20-item questionnaire (see below for details of its development and validation) to determine if they are experiencing any motor or nonmotor PD symptoms or if there are any signs of atypical parkinsonian disorders. They will also undertake objective tests to measure their performance on specific motor tasks, comprising spoken voice, tremor, dexterity, and mobility (see below for details of their selection and validation). Tests will be performed, and data collected, using mobile smartphone technology.

Data collected via the digital platform is stored in a database created and managed by the Thai Red Cross Society, strictly in accordance with the relevant Institutional Review Board and Personal Data Protection Act regulations. The Thai Red Cross Society is a major national non-profit public organization that undertakes humanitarian activities, including those related to the promotion of quality of life and disease treatment and eradication, in keeping with the principles of the International Red Cross. The Society is closely affiliated with ChulaPD, the only tertiary center in Thailand dedicated to the study and care of PD, with whom they have collaborated over many years on national projects related to PD and its management (28, 107).

Identification of individuals who may have undiagnosed PD (both early and more advanced), those who may be in the prodromal stage, or those at risk of developing the condition will be evaluated by different machine learning models using combined data from the questionnaire and objective measurements.

### 2.2 Questionnaire development and validation

The Parkinson’s disease risk survey questionnaire was developed and validated by an Expert Panel, consisting of movement disorder neurologists, Parkinson’s disease nurse specialists, and representatives from a PD support group (patients and caregivers). The questionnaire forms part of the Parkinson’s disease risk survey digital platform and participants complete it using a mobile smartphone device with responses input using the touch screen. Table 2 shows the content of the questionnaire translated into English. The original Thai version of the questionnaire can be found in Supplementary material 1. Details of questionnaire development and validation are described below.

**Table 2:** Parkinson’s disease screening questionnaire (English version).

	Yes	No
Have you ever noticed a tremor in your hands or legs at rest?		
Have you started writing slower or has your handwriting changed or become smaller?		
Have you noticed that you have become more clumsy or slower in any tasks: for example, combing hair, dressing, or bathing, or have more difficulty with tasks that involve fine hand motor control, such as doing up your buttons or opening a bottle?		
Have you noticed that your voice has become softer or more monotonous or that listeners have to ask repeatedly because they cannot hear you speak?		
Have you noticed that your arms swing less or do not swing when you walk?		
Do you walk with short steps or a shuffling gait?		
Do you have difficulty walking or freeze when starting to walk, while walking, or when turning?		
Do you have trouble with throwing yourself forward while walking causing you to keep up with your step or difficulty if you need to stop walking immediately?		
Do you have any of the following problems? Difficulty turning over when sleeping, getting out of bed or getting up after sitting down.		
Does your tremor, slowness, or stiffness start on one side of your body first?		
Do you know if you have symptoms of speaking, shouting or moving your arms and legs while dreaming or have you fallen off the bed while sleeping? Or have you ever been told by a bed partner or caregiver that you have these symptoms?		
Do you usually have excessive sleepiness during the day or fall asleep while doing an activity?		
Do you feel that your sense of smell has decreased?		
In the past 3 months, have you had chronic constipation, defined as defecating less than 3 times/week?		
Do you have symptoms of depression, for example, crying more easily than usual or a lack of interest in the surrounding environment or things that used to be fun in the past?		
Have you ever seen a hallucination or heard a sound without a person being present?		
Do you usually feel lightheaded or dizzy when changing position from supine or sitting to standing up, and do these symptoms usually improve or disappear after sitting or lying down?		
Do you usually have urinary control problems, such as being unable to control urine, urinary incontinence, or urinary retention?		
Do you have problems with analytical thinking, memory, or calculation that has worsened over more than 1 year?		
Do you have balance problems or frequent falls at the very beginning of experiencing tremor, slowness, or stiffness?		

#### 2.2.1 Format

The Panel agreed that the survey should capture basic demographic data, information on the presence of, or exposure to, any potential risk factors or protective factors, and ask questions about motor and nonmotor PD symptoms, as well as potential atypical parkinsonian symptoms, that the individual might be experiencing. In terms of structure, focus and length, for the symptoms section of the survey, it was agreed that a total of 20 questions would be optimal to ensure participant engagement and completion of the survey. Questions are answered either “yes” or “no” and subjects’ responses are coded as “1” (yes) and “0” (no). Questions 1–10 relate to the presence of motor symptoms, questions 11–15 relate to the presence of nonmotor symptoms, and questions 16–20 determine the presence of “red flags” suggesting that the subject may have atypical parkinsonian disorders.

#### 2.2.2 Feature selection

Screening questions for motor symptoms (questions 1–10) were developed based on the MDS clinical diagnosis criteria for PD (108) and questions on prodromal/nonmotor symptoms on the MDS Research Criteria for Prodromal Parkinson’s Disease (59). Symptoms and signs previously considered in PD and parkinsonism screening questionnaires from previous community and hospital-based PD screening questionnaire development studies were also comprehensively reviewed (109–112). The Panel then reached a consensus on those items that were likely to indicate the presence of prodromal PD most strongly (63, 64, 109, 111, 113, 114).

In terms of motor symptoms, tremor and shaking, bradykinesia and troublesome arm swinging, stiffness, gait and posture, and difficulty getting up after sitting down were good questions to discriminate between PD and healthy controls according based on sensitivity and specificity. The sensitivity and specificity, respectively, of tremor and shaking were: 85.8% (95% CI: 79.1–90.7) and 89.9% (95% CI: 82.3–94.6), bradykinesia and troublesome arm swinging: 72.7% (95% CI: 64.7–79.5) and 98.1% (95% CI: 92.6–99.7), stiffness: 88.9% (95% CI: 82.5–93.2) and 80.0% (95% CI: 71.1–86.8), gait: 69.0% (95% CI: 61.0–76.1) and 91.2% (95% CI: 84.6–96.0), posture: 59.4% (95% CI: 51.2 67.1) and 92.7% (95% CI: 85.7–6.6), difficulty getting up: 42.5% (95% CI: 34.6–50.7) and 82.7% (95% CI: 71.4–89.0). The combination of bradykinesia and slowness, gait and posture, and difficulty getting up was 87.5% and 86.3%, respectively. In a community survey, a problem was identified with question 9 relating to the phrase “difficulty getting up after sitting down” due to orthopedic problems in some respondents, so an additional question regarding “difficulty rising from bed or turning in bed” was added to question 9.

The five prodromal/nonmotor symptom questions (questions 11–15) were based on the published order of likelihood ratio (LR) (59, 65) and included hyposmia (LR 6.4), possible RBD based on questionnaire responses (LR 2.8), excessive daytime sleepiness (LR 2.7), constipation (LR 2.5), and depression (LR 1.6).

The five questions on “red flag” items relating to atypical parkinsonian disorders (questions 16–20) were derived from a review by the Expert Panel of the main symptoms that discriminate PD from the four main atypical parkinsonian disorders namely progressive supranuclear palsy, corticobasal syndrome, multiple system atrophy, and dementia with Lewy bodies, based published differential diagnostic criteria for these conditions (115–120) along with the red flags criteria of the MDS clinical diagnosis criteria for PD (108).

#### 2.2.3 Validation

There are various different approaches that can be used for the assessment of content validity; however, a review of the published literature suggests that there is no agreed consensus on the best method for patient-focused instruments (121, 122). Some studies use Expert Panel opinion only without any grading while others propose 3-, 4-, or 5-item grading systems. In our study, the content validity of the symptom questionnaire was initially evaluated by the Expert Panel for both its relevance to the clinical presentation of prodromal and early PD, and its applicability to the Thai population. We also used a standard 3-level anchor system in rating the questionnaire, as used successfully in other PD questionnaire studies (114). Each item was graded in three levels (0 = not relevant, 0.5 = moderately relevant, 1 = very relevant) (114). The content validity was calculated as the mean score of all items with any mean score > 0.5 being considered acceptable. Using our chosen method, the content validity for the questionnaire was a mean ± standard deviation (SD) of 0.97 ± 0.07 (range: 0.85–1.0).

The reliability of the questionnaire was tested on a cohort of 15 people with PD (7 male, 8 female; mean [± SD] age: 67.6 ± 9.7) and 15 control subjects (7 male, 8 female; mean [± SD] age: 51.8 ± 9.0) without PD using Cronbach’s alpha coefficient of the total score. A Cronbach’s alpha coefficient > 0.7 was considered as indicating good reliability. The Cronbach’s alpha coefficient for the questionnaire was calculated to be 0.939. Test–retest reliability was evaluated in a cohort of 100 people with PD (54 male, 46 female) with 3 months between the first and second tests. Demographic characteristics of the test–retest study participants are summarized in Table 3. Significant test–retest reliability was confirmed for all 20 questions (*p* < 0.001 in each case). Pearson’s correlation between PD and the total sum score of the 20-item questionnaire was R = 0.931 (*p* < 0.001). The Pearson’s correlation for each individual item is shown in Supplementary material 2. The receiver operating characteristic (ROC) curve for PD classification using the symptoms questionnaire with 10-fold cross validation which showed a mean area under the curve (AUC) of 0.92 ± 0.02.

**Table 3:** Demographic characteristics of subjects who participated in the questionnaire test–retest validation.

Parameters	Male (*n* = 54)	Female (*n* = 46)	*p*-value (Chi-squared test)
Age, years (mean ± SD)	62.0 ± 14.8	56.1 ± 10.2	0.030^*^ (Unpaired *t*-test)
Status, %	Single 21.4%	Single 25.5%	0.933
	Married 66.7%	Married 61.8%	
	Divorced 4.8%	Divorced 3.6%	
	Unknown 7.1%	Unknown 9.1%	
History of drinking alcohol, %	No 47.6%	No 90.9%	<0.001^*^
	Yes 50.0%	Yes 9.1%	
	Quit 2.4%	Quit 0%	
History of drinking coffee, %	No 33.3%	No 40.0%	0.532
	Yes 67.7%	Yes 60.0%	
History of drinking milk, %	No 42.9%	No 30.9%	0.287
	Yes 57.1%	Yes 69.1%	
History of smoking, %	No 73.8%	No 96.4%	0.005^*^
	Yes 9.5%	Yes 1.8%	
	Quit 16.7%	Quit 1.8%	
History of consuming dairy products, %	No 66.7%	No 52.7%	0.213
	Yes 33.3%	Yes 47.3%	
History of undertaking physical exercise, %	No 38.1%	No 74.5%	<0.001^*^
	Yes 61.9%	Yes 25.5%	
History of pesticide exposure, %	No 97.6%	No 89.1%	0.135
	Yes 2.4%	Yes 10.9%	
Previous head injury, %	No 92.9%	No 98.2%	0.313
	Yes 7.1%	Yes 1.8%	
History of drug addiction, %	No 100%	No 100%	NA
Concomitant diseases, %	No 52.4%	No 43.6%	0.419
	Yes 47.6%	Yes 56.4%	
Diabetes mellitus	26.2%	23.6%	0.815
Hypertension	28.6%	23.6%	0.634
Dyslipidemia	4.8%	7.3%	0.695
Heart disease	4.8%	0%	0.185
Cerebrovascular disease	4.8%	1.8%	0.577
Gout	2.4%	0%	0.433
Benign prostatic hypertrophy	7.1%	0%	0.078
Knee osteoarthritis	0%	7.3%	0.131
Gastritis	2.4%	5.5%	0.631
Other	4.8%	18.2%	0.063

SD, standard deviation; ^*^statistically significant difference between groups at the *p* < 0.05 level.

### 2.3 Digital marker selection and validation

**Figure 4:**
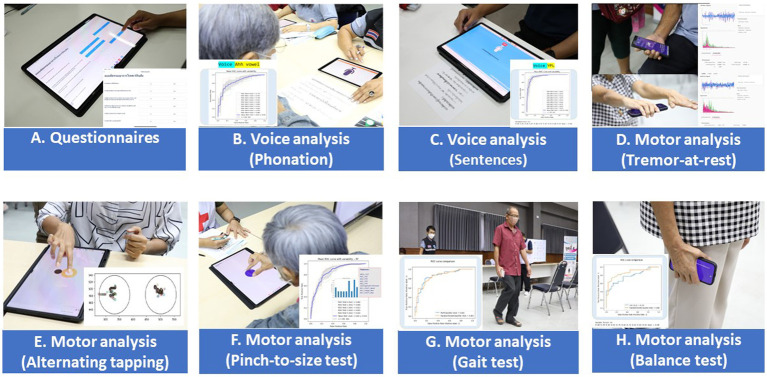
Application interface of the digital assessment package and how the assessment was undertaken.

The Expert Panel reviewed potential early diagnostic markers of PD with the aim of creating a set of digital markers suitable for large volume screening of the general population and which could be delivered using the digital platform (Figure 4).

#### 2.3.1 Digital marker selection

Digital markers identified for inclusion in the Parkinson’s disease risk survey were selected following a review of published studies that have used mobile devices and machine learning tools to quantify PD (83–85, 87). These studies have shown that using a combination of tests with machine learning-based analysis it is possible to distinguish subjects with PD from controls with a high degree of sensitivity and specificity. Common testing activities for PD diagnosis using smartphone applications include gait, voice, finger tapping, memory tests and questionnaires. For objective testing of motor function, the Parkinson’s disease risk survey will use a smartphone application to collect data on spoken voice, tremor, dexterity, and mobility. All selected markers are ones that ChulaPD has considerable research experience with. The final digital marker selection includes eight variables, as described in Table 4: voice vowel, voice sentences, resting and postural tremor, alternate finger tapping, “pinch-to-size” test, and gait and balance tests. The rationale for these selections and their applicability to the digital platform are summarized below.

**Table 4:** The eight selected digital markers and the number of recordings undertaken for each test activity during validation.

Activity	Subjects with PD	Control subjects
	Number of test recordings for each activity	
Voice vowel	315	183
Voice sentences	512	331
Resting tremor	211	154
Postural tremor	211	154
Alternate finger tapping	324	205
Pinch-to-size	294	205
Gait	280	202
Balance	284	201

PD, Parkinson’s disease.

##### 2.3.1.1 Voice vowel and voice sentence tests

Difficulty in speech, known as dysarthria, is reported to be one of the earliest symptoms of PD, specifically involving degraded phonation, articulation, and prosody (123–126). Voice assessment is one of the most discriminatory features of PD and its changes may be the first motor sign in the prodromal phase (127, 128). To evaluate dysarthria, individuals typically undergo assessments that involve utilizing sustained-vowels, words, or sentences. Features extracted from voice data in the frequency domain include fundamental frequency, pitch, and harmonics, while features extracted in the time domain consist of amplitude, period, jitter, and shimmer. Singh and Xu (92) implemented several machine learning classification approaches on voice samples of individuals saying “Aaah” for 10 s from mPOWER dataset to differentiate between PD and non-PD cases. The authors reported a classification AUC of 0.99 when using L1-based feature selection in conjunction with SVM. Similarly, Despotovic et al. (91) achieved a classification accuracy of 96.92% by applying the gaussian processes Automatic Relevance Determination (ARD) to identify individuals with PD using sustained vowels from the Parkinson’s Telemonitoring dataset. Several studies have reported that features extracted from sustained vowels recordings are more effective in discriminating between PD and non-PD cases than features extracted from words or sentences (91, 129, 130). However, several studies have reported that using sentences is more effective than using sustained vowels for classification, with accuracy ranging from 82% to 99%, as sentences provide more insights into prosody, phonation, articulation (123, 131, 132).

While motor tests such as tremor, gait and finger tapping are applicable regardless of the individual’s language, in the case of speech tests, the subject may be unable to understand the test or complete it successfully due to challenges with pronunciation, which may influence the test results and their interpretation (133). To overcome this, the voice tests used for Parkinson’s disease risk survey digital platform implemented in Thailand comprise newly developed speech tests that use sustained vowels (making a loud “Aah” sound for 10 s to assess phonation) and speaking sentences in Thai language (to assess prosody and articulation), thereby enhancing its relevance for Thai native speakers.

##### 2.3.1.2 Resting and postural tremor tests

Tremor is an involuntary, and often rhythmic, shaking of various parts of the body with one of the most common sites in subjects with PD being the hands. The types of tremors commonly used to test for a PD diagnosis are “rest” and “postural” tremor. A rest tremor involves placing the hands in a relaxed position, e.g., resting hands in the lap, with no intention of movement (134). Postural tremor describes tremor that occurs while maintaining a posture (135). Data obtained from a smartphone’s inertial sensors can be used to quantify the level of tremor (93), thereby helping to determine whether the person has PD. Features from tremor data are extracted in both the time and frequency domains. In the time domain, these features include temporal fluctuation, root mean square (RMS) of linear acceleration, and approximate entropy. In the frequency domain, they encompass power frequency and power spectral density. Surangsrirat et al. (94) reported 100% classification accuracy, sensitivity, and specificity using SVM as a classifier, tested with 10-fold cross-validation, and utilizing only the temporal fluctuation feature for classification.

##### 2.3.1.3 Alternate finger tapping and “pinch-to-size” test

Impaired dexterity has been documented as one of the earliest motor signs in PD and can be demonstrated about 6 years prior to the diagnosis of PD (46). Abnormal dexterous movements can be detected by alternating two fingers tapping on the computer keyboard or on the smartphone (96, 136, 137). Studies have shown that the machine learning approach using the various parameters of finger tapping tests can discriminate PD from controls with good accuracy, suggesting that the alternating finger tapping test would be a promising digital marker to quantify subtle motor symptoms in early PD (96).

A finger pinching test, referred to in this study as the “pinch-to-size test,” will also be used to supplement the finger tapping test. This test was designed to detect PD tremor in fingers and hands by collecting screen touching data while subjects pinch their thumb and index finger to track the size of a circle on a mobile phone screen. The pinch-to-size test is similar to the finger tapping test, where subjects are asked to tap their index finger on the same hand repeatedly as fast as possible and move the index finger away from the thumb as far as possible after tapping (138, 139). This finger tapping test is normally used to assess the finger rigidity. The pinch-to-size test selected for the Parkinson’s disease risk survey assesses finer movement as well as the hand-eye coordination of both fingers because the pinching speed and size are constrained by the target circle’s shape. The pinch-to-size test is also convenient to perform, as it resembles the pinching movement used on a mobile phone screen to zoom in or out.

##### 2.3.1.4 Gait and balance tests

People with PD can experience gait and balance disturbances, which may appear as slow walking, shuffling, dragging steps, and struggling to start or stop walking (140, 141). If combined with an unstable posture, the individual will be more susceptible to the risk of falling. The accelerometer and gyroscope functions of a smartphone can be used to quantify gait patterns. Features extracted from gait data are acceleration amplitude, gait speed, stride intervals, peak frequency, and wavelet bands. Pittman et al. (89) attained an accuracy of 92% in classifying PD and non-PD individuals using a decision tree and artificial NN using data collected during a walking activity (10 s walking and 10 s standing still). Similarly, Zhang et al. (90) employed deep neural network as the classifier, with the authors reporting an area under the receiver operating characteristic curve (AUC) of 0.87.

#### 2.3.2 Digital marker validation and data analysis

The eight selected markers have been developed into a digital application that has been validated as described below. Validation was undertaken in a cohort of 343 people with PD (48.6% male) and 230 control subjects (27.9% were male) without PD. Participants undertook each test activity using a mobile application and data were recorded using the built-in sensors of the mobile phone, namely the accelerometer, gyroscope, microphone, and touch screen. The validation study was approved by the Human Ethics Committee of the Faculty of Medicine, Chulalongkorn University (IRB number 0804/65). All subjects gave written informed consent before the enrolment into the study, in accordance with the declaration of Helsinki. Performance on the eight test activities were recorded by each participant using a mobile application and the built-in sensors of a smartphone, namely the accelerometer, gyroscope, microphone, and touch screen. Details of how each testing activity was performed is described in Table 5.

**Table 5:** Performance methods for each of the digital marker test activities.

Marker	Description of test
Voice vowel	Participants pronounce the sustained vowel /a/, as in “*bar*,” for 10 s without holding their breath. The test is repeated three times for each participant.
Voice sentences	Participants read sentences aloud in the Thai language, such as the example sentence “*rod tid mak loey*,” which means “*The traffic is really jammed*.” The test includes a total of 25 sentences that are commonly encountered in real-life situations.
Resting tremor	Participants are instructed to place the mobile phone on one palm with the screen facing up and lay their hand in their lap. They are then asked to specify whether the device is on the left- or right-hand side by pressing on the screen. Finally, participants are instructed to keep their hand still, along with the device, for 20 s.
Postural tremor	Participants are instructed to hold the device and press on the screen to verify which side of the hand they are holding, then stretch out their arm and maintain that posture for 20 s.
Alternate finger tapping	Participants place the device on a flat surface. They are then instructed to use their index and middle fingers of one hand to tap on two circles that appear on the screen, alternating between the two fingers as quickly as possible while maintaining correct finger alternation within 10 s.
Pinch-to-size	Participants place the device on a flat surface. They are instructed to use their thumb and index finger of one hand to pinch, following the varying size of a circle that also moved randomly across the screen. The test is repeated five times for each participant.
Gait	Participants hold the device and walk in a straight line for 3 meters, then turn around and walk back to the starting position. The route for testing is set up to indicate the walking direction and the turning point.
Balance	Participants are instructed to put the device in a pocket after pressing start to begin the test. They then stand still for 30 s. A tone is played to signify the end of the test.

##### 2.3.2.1 Feature extraction

Features were extracted using Python SciPy library (142). SciPy.stats was used for statistical parameters (e.g., mean, SD, kurtosis, and skew). SciPy.signal and SciPy.fft were used for extracting time (e.g., period amplitude) and frequency domain features (e.g., harmonics and formants). The extracted features were labeled according to the participant’s group before feeding to the machine learning classifier.

##### 2.3.2.2 Choice of machine learning classifier

Many studies have demonstrated the effectiveness of using machine learning classification algorithms to accurately differentiate individuals with and without PD based on data collected through smartphone applications.

Initially, the performance of three machine learning classifiers was compared: multilayer perceptron (MLP), SVC, and random forest (RF) using the hold-out method for dataset splitting, to find which would be best suited for the Parkinson’s disease risk survey data. We used the default settings for each classifier from scikit-learn (143) version 1.3.0. The RF algorithm was chosen because it demonstrated higher accuracy in classifying PD compared to SVC and performed comparably to MLP in our preliminary analysis using tremor and gait recordings. The superior performance of the RF algorithm compared to SVC and MLP aligns with the findings of Sarkar et al. (144).

RF is a machine learning algorithm composed of an ensemble of decision trees. In a classification task, each branch of the decision tree is trained using subsets of sampled training data labeled by the predicting classes. During the learning process, decision trees perform a series of comparisons on selected input features (node splitting) until a predicting class is assigned to the feature. The final predicted class of the RF is determined by the majority of the output classes from the decision trees.

In the validation study, an RF classifier was used to predict whether activity recordings were from a person with or without PD. The forest consisted of 100 trees, and Gini impurity was used to measure the quality of splits. The maximum number of features was determined by the square root of the total number of features before searching for the best node splitting. Ten-fold cross-validation was employed to assess the generalizability of the classifiers. In each test activity, the data was divided into 90% for training and 10% for testing sets. Classifiers trained specifically for a type of activity are referred to as specialized classifiers.

##### 2.3.2.3 Ensemble learning

The validation study dataset, as with many smartphone-based studies, contains missing data. Many participants only completed subsets of available tests, with less than half completing all available tests. In addition, PD symptoms are heterogeneous, meaning that patients can manifest different onset and progression patterns. To address these challenges, an ensemble classifier voting approach was employed. With this approach, the classifiers differ only in the training set based on the test activity, while the algorithm remains the same (creating a homogeneous ensemble).

Previous studies have demonstrated the benefits of accounting for the intersubject heterogeneity of PD by either combining the activity data or classifiers to enhance classification accuracy. Schwab and Karlen (145) introduced an evidence aggregation model (EAM) that integrates four trained NN models specialized in detecting PD for each test activity (walking, voice, finger tapping, and memory). This approach aims to combine several model outputs and generate a single diagnostic score. The EAM achieved an AUC of 0.85, surpassing the PD classification achieved by a single activity with single classifiers. Prince et al. (97) implemented a multi-source ensemble learning approach that utilizes three test activities (walking, voice, and finger tapping) with combined classifiers, including single neuron, random forest, convolutional NN, and deep NN. The ensemble approach classification accuracy was 82%, outperforming single activity with single or combined classifiers for PD classification. Therefore, an ensemble approach will be used for the Parkinson’s disease risk survey data analysis.

**Figure 5:**
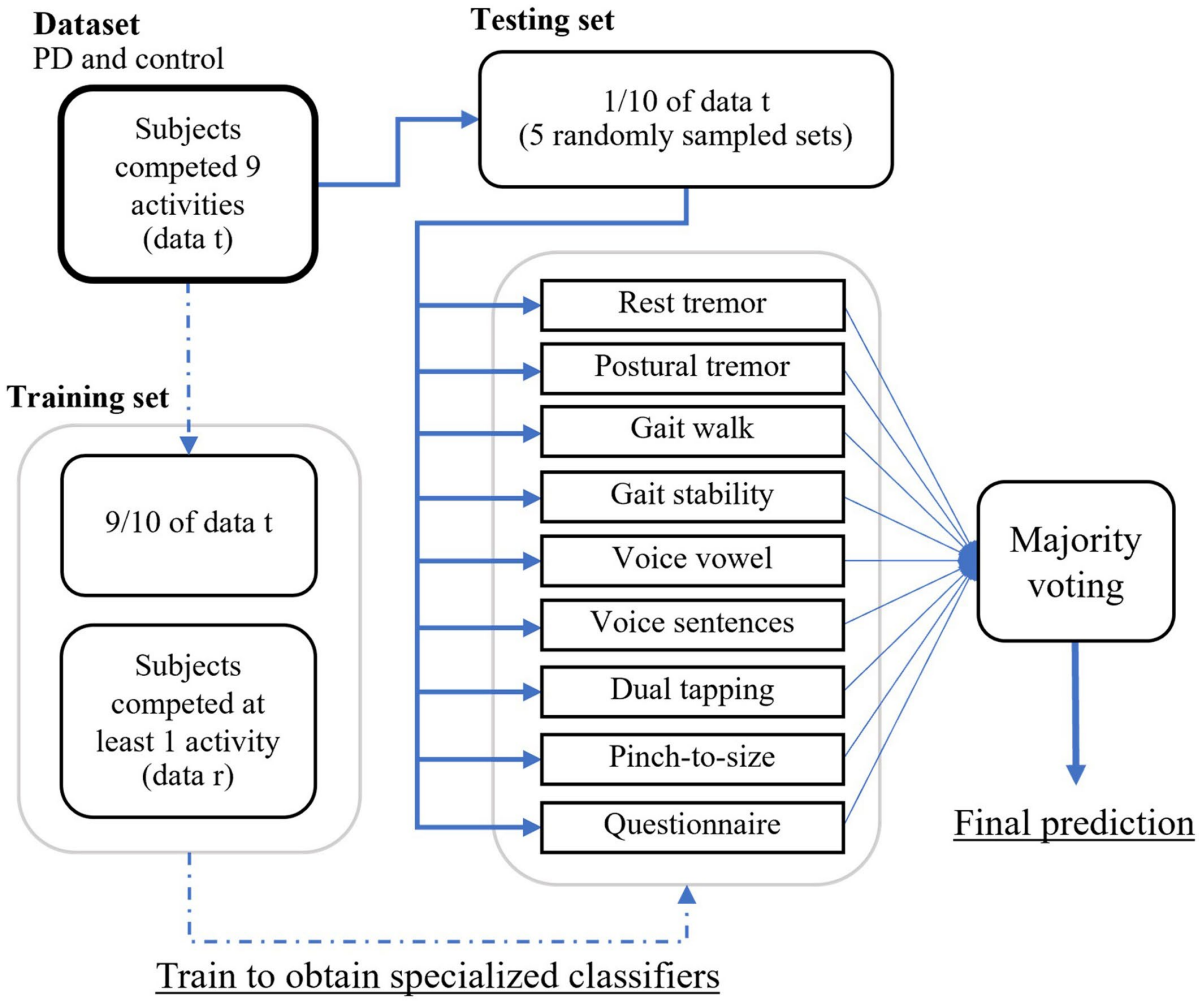
The procedure for splitting the training and testing sets for PD classification using ensemble learning.

The procedure for splitting the training and testing sets for PD classification using ensemble learning is illustrated in Figure 5. Of the 343 people with PD, 162 completed all tests, while among the 230 individuals without PD, 128 completed all tests. Data from subjects who completed all tests (denoted as data t) were divided into a 90% training set and a 10% test set. The training set was combined with the data from subjects who did not complete all tests (denoted as data r) to train a specialized model for each test.

A randomly sampled subset of the test set was used to predict with specialized models. This process was repeated five times for each combination of activity sets. The optimal combination of activity sets was selected based on each participant’s available recorded activities. Model outputs were then subjected to majority voting for the final classification as either PD or non-PD.

##### 2.3.2.4 Classification metrics

The performance of specialized and ensemble classifiers in distinguishing between PD and non-PD was assessed using the area under the receiver operating characteristic (ROC) curve (AUC) (146). The ROC curve illustrates the true positive rate and false positive rate at different classification thresholds, with PD assigned as the positive class and non-PD as the negative class. The true positive rate represents the ratio of correctly predicted PD cases to the total number of actual PD cases, while the false positive rate reflects the ratio of incorrectly predicted PD cases to the total number of actual non-PD cases. AUC calculations were performed using the “sklearn.metrics” function in Python’s scikit-learn library, version 1.3.0.

The true positive rate (TPR) and true negative rate (TNR) were calculated using Equations 1, 2 to demonstrate the proportion of correct predictions for each class by the ensemble classifiers.


(1) $$TPR=\frac{TP}{TP+FN}\#$$

(2) $$TNR=\frac{TN}{TN+FP}\#$$

Where TP (true positive) is the number of correctly PD cases, FN (false negative) is the number of PD cases incorrectly predicted as non-PD, TN (true negative) is the number of correctly predicted non-PD cases, and FP (false positive) is the number of non-PD cases incorrectly predicted as PD.

The accuracy, Equation 3, calculated as the number of correctly predicted PD cases divided by the total number of predictions, is as follows.


(3) $$\mathrm{Accuracy}=\frac{TP+TN}{TP+TN+FP+FN}\#$$

The precision-recall (PR) curve was specifically used to compare PD classification when using voice vowel and voice sentences. This examination aimed to assess whether the data imbalance between PD and non-PD has an impact on classifier performance in this metric compared to the ROC curve (147). For instance, classifiers may predict PD accurately but perform poorly in predicting non-PD cases. The PR curve was calculated using the “sklearn.metrics” version 1.3.0.

##### 2.3.2.5 Validation results

Table 6 summarizes PD classification performance using classifiers specialized for individual activity data, measured by the mean AUC with 10-fold cross validation. PD classification using balance data achieved the highest mean (±SD) AUC of 0.88 ± 0.06. The use of voice vowels resulted in a mean (±SD) AUC of 0.82 ± 0.04, which was the lowest value.

**Table 6:** Mean area under the receiver operating characteristic curve after 10-fold cross validation for each test activity.

Activity	Mean AUC ± SD
Balance	0.88 ± 0.06
Postural tremor	0.87 ± 0.06
Dual tapping	0.86 ± 0.05
Pinch-to-size	0.85 ± 0.05
Gait	0.84 ± 0.07
Questionnaire	0.83 ± 0.04
Voice sentences	0.83 ± 0.04
Rest tremor	0.83 ± 0.06
Voice vowel	0.82 ± 0.04

Test activities are sorted by their mean area under the curve (AUC) in descending order.SD, standard deviation.

**Figure 6:**
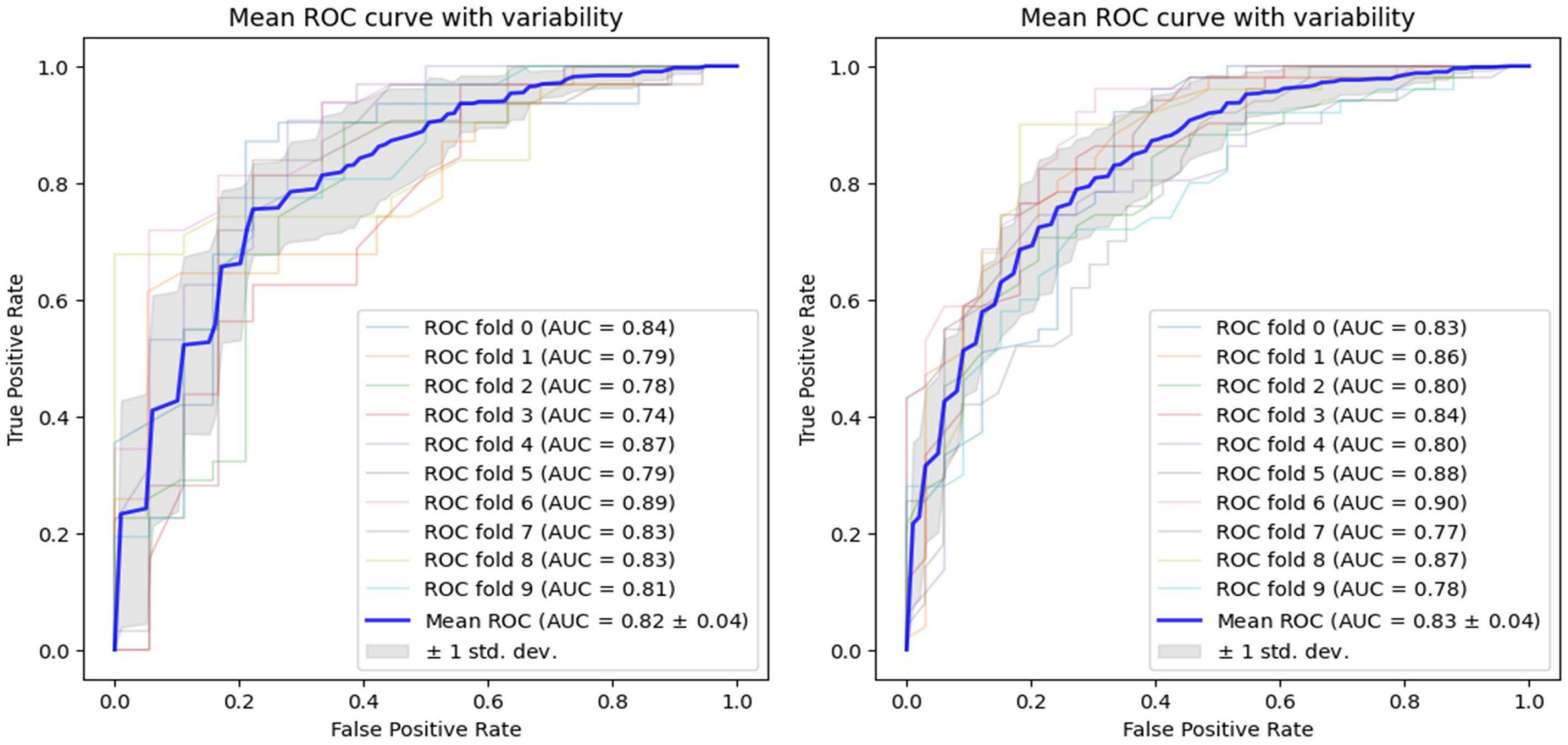
Receiver operating characteristic curve of classifiers using voice vowel data (left) and voice sentence data (right).

**Figure 7:**
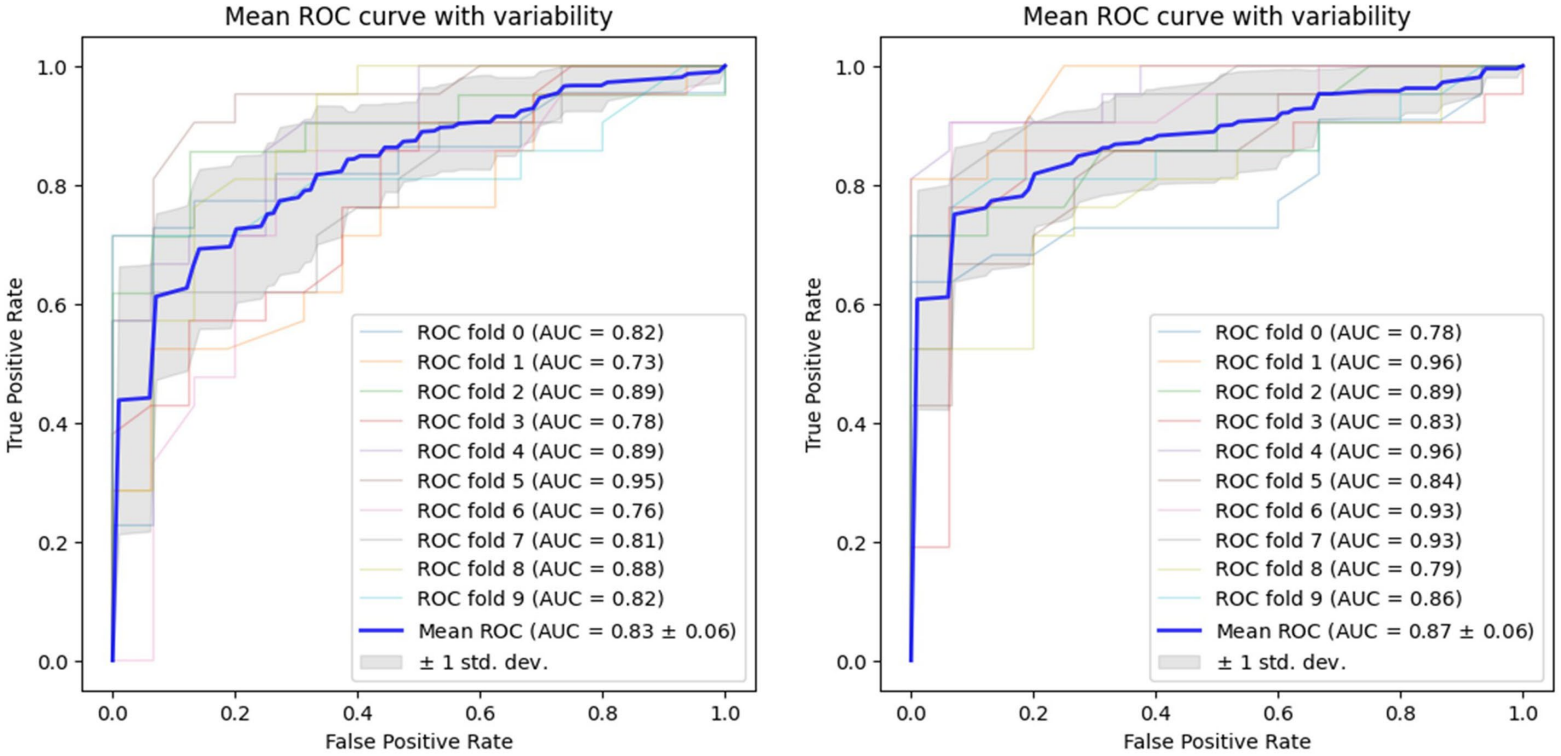
Receiver operating characteristic curve of classifiers using rest tremor data (left) and postural tremor data (right).

**Figure 8:**
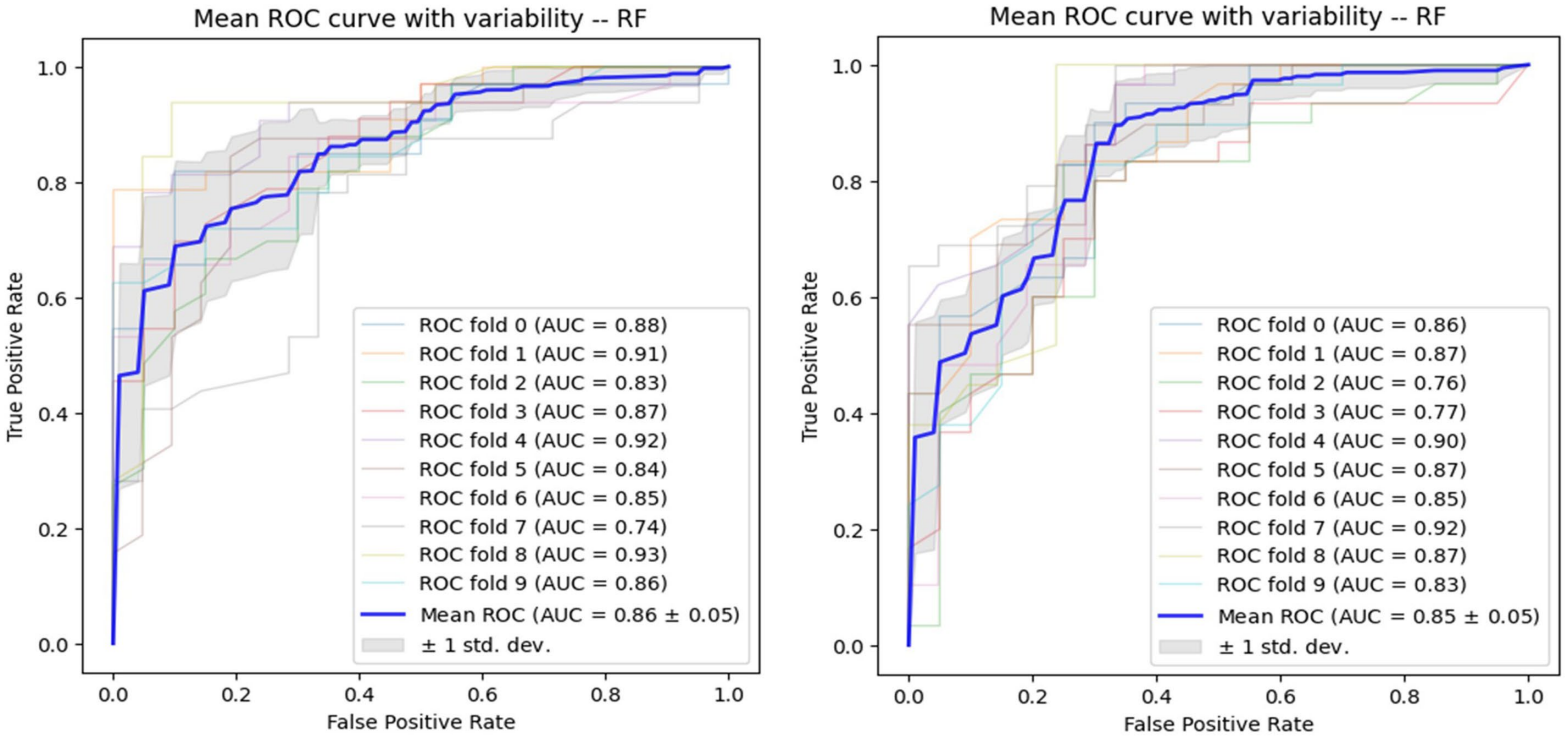
Receiver operating characteristic of classifiers using alternate finger tapping data (left) and pinch-to-size data (right).

**Figure 9:**
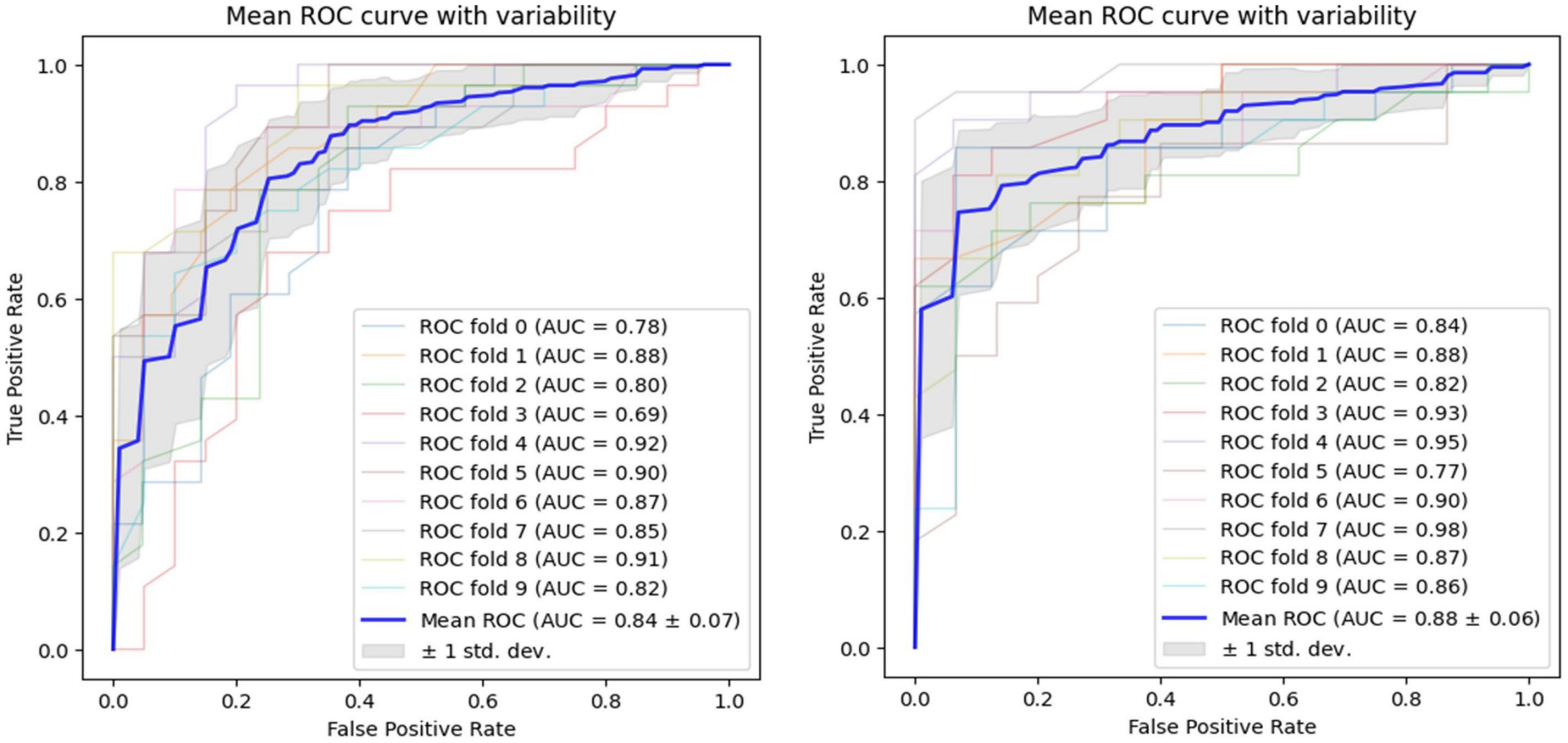
Receiver operating characteristic of classifiers using gait data (left) and balance data (right).

Using voice vowel and voice sentence data ROC curves for PD classification using RF with 10-fold cross validation showed mean (±SD) AUC values of 0.82 ± 0.04 and 0.83 ± 0.04, respectively (Figure 6). Using resting tremor data and postural tremor data, ROC curves for PD classification using RF with 10-fold cross validation showed mean (±SD) AUC values of 0.83 ± 0.06 and 0.87 ± 0.06, respectively (Figure 7). Using dual tapping and pinch-to-size data, ROC curves for PD classification using RF with 10-fold cross validation showed mean (±SD) AUC values of 0.86 ± 0.05 and 0.85 ± 0.05, respectively (Figure 8). Using gait and balance data, ROC curves for PD classification using RF with 10-fold cross validation showed mean (±SD) AUC values of 0.84 ± 0.07 and 0.88 ± 0.06, respectively (Figure 9).

Overall, the AUC values of the specialized classifiers ranged from 0.82 to 0.88. Balance testing achieved the highest AUC of 0.88. The ensemble classifier, which utilized a combination of questionnaire responses, voice vowels, rest tremor, gait and balance activities achieved a PD prediction accuracy of 0.9108 (Table 7). Taken together, these results confirmed the validity of the selected digital markers and the mobile application and testing system for distinguishing between subjects with and without PD.

**Table 7:** The top eight test activity combination sets achieved the highest mean accuracies when evaluated on five randomly sampled test sets.

Activity count	Activities									Accuracy of 10-fold CV test set randomly selected 5 times	
	Questionnaire	Voice sentence	Voice vowel	Postural tremor	Rest tremor	Gait	Balance	Dual tap	Pinch-to-size	Mean	SD
5	✓		✓		✓	✓	✓			0.9108	0.012357
8	✓	✓	✓	✓	✓	✓	✓		✓	0.9106	0.006148
8	✓	✓	✓	✓		✓	✓	✓	✓	0.9094	0.011908
5	✓	✓	✓	✓			✓			0.9094	0.006285
8	✓		✓	✓	✓	✓	✓	✓	✓	0.9046	0.010918
7	✓		✓	✓	✓	✓	✓		✓	0.9042	0.009391
8	✓	✓		✓	✓	✓	✓	✓	✓	0.9032	0.013221
7	✓		✓	✓		✓	✓	✓	✓	0.9018	0.008849

Activity count indicates the number of specialized classifiers used for ensemble learning.CV, cross validation; SD, standard deviation.

## 3 Discussion

This article outlines the protocol for the Parkinson’s disease risk survey in Thailand which will implement screening of the national population for early signs of PD using a digital platform. Results of validation tests have demonstrated both the validity and reliability of the digital platform that comprises a symptom questionnaire and objective tests of a range of motor tasks, for population screening in both community and hospital settings, and its ability to discriminate between people with and without signs of PD. The first field study was undertaken in Nakhon Sawan Province in January 2024 and the survey is being rolled out throughout Thailand to all citizens over the age of 40 years. Data collection from individual citizens, from further provincial field tests, and from hospital screening stations is ongoing. Data collected from the survey will be used to identify subjects who have either prodromal PD or early signs of clinical PD.

Identification of strategies that could slow the human aging process, and thereby reduce the occurrence and impact of the many chronic noncommunicable diseases, such as PD, associated with advanced age, would undoubtedly have huge societal benefits, and have therefore been the focus of considerable research.

Lifestyle interventions that have been evaluated for their positive benefits in both health and disease prevention include exercise, dietary modifications and sleep. Looking at the available evidence for the benefits of these interventions, in healthy subjects, an 8-week treatment program that included diet, exercise and sleep guidance was shown to have a positive impact on parameters of aging (148). Analysis of patterns of DNA methylation, a recognized biochemical marker of age, in participants was used to calculate their DNAmAge. The program of lifestyle interventions was associated with a statistically significant 3.23-year decrease in subjects’ DNAmAge compared with controls, a potential reversal of epigenetic age.

In the neurology field, various lifestyle interventions have been assessed for their ability to prevent or slow the progression of dementia, and to reduce the risk of developing Alzheimer’s disease and other conditions, including PD, that have protein accumulation pathology (149, 150). A systematic literature review that evaluated studies of multidomain lifestyle interventions (diet, physical activity and cognitive training) on cognitive outcomes in older adults, including those at risk of dementia found that these strategies had beneficial effects in preventing or delaying cognitive decline (151). In the case of PD, it is suggested that for patients identified in the prodromal or very early stage of PD, relatively simple lifestyle modifications that focus on consuming a healthy diet (EAT), increasing engagement with exercise (MOVE), and getting sufficient good quality sleep (SLEEP), might have a more beneficial and timely effect in terms of overall outcomes than waiting to treat established disease (12). Although such interventions rely heavily on patient adherence, they are also relatively risk-free and easy to implement.

Those subjects identified in this first phase of the national screening program as having prodromal PD, will subsequently be targeted in the second phase with EAT, MOVE, SLEEP lifestyle interventions on an individual case-by-case basis under the direction of a neurologist alongside monitoring of intermediate and longer-term outcomes. To achieve this on a national scale, we plan to utilize the existing Thai Red Cross Society network, which is available in all provinces in Thailand to facilitate implementation and monitoring of interventions. This approach was successfully applied by the ChulaPD team for the deployment of a laser cane for PD patients with freezing of gait. Using this collaborative network, it was possible to distribute laser canes to patients all over Thailand, achieving benefits in terms of Social Return on Investment of 2.17 (105, 152). However, it is recognized that implementing any such interventions in real-life, population-based settings is associated with many challenges, not least subject adherence to the proposed program, and each intervention type has its pros and cons depending on individual circumstances (153). In addition, it is likely that the implementation process for lifestyle interventions will need to be adapted to local needs and the situation in individual provinces.

Evidence of the benefits of each of the aforementioned lifestyle interventions in neurological diseases is summarized below.

### 3.1 Eat

Various different dietary styles and the use of nutritional supplements have been investigated for their potential to slow the development and progression of PD, and as part of the management of established disease, although data are primarily from observational studies (154–156). There has been increasing interest in “food as medicine” and how dietary components, in particular plant-derived compounds, might attenuate dopaminergic neuron degeneration, reduce α-synuclein aggregation, and modulate neuroinflammatory responses in people with PD (157, 158). A study undertaken in Greece assessed the probability of prodromal PD in a population-based cohort of older adults using MDS research criteria and investigated its possible association with consumption of a Mediterranean diet (159). This dietary style focuses on consumption of high fiber vegetables, fruits, herbs, nuts, beans and whole grains, with moderate amounts of dairy, poultry, eggs and seafood, but a low intake of red meat. A significantly lower probability for prodromal PD was observed in subjects who had high scores for adherence to a Mediterranean diet, and this was related primarily to nonmotor markers, such as depression, constipation, urinary dysfunction, and daytime somnolence. Notably, subjects in the highest adherence quartile had a 21% lower probability of prodromal PD than those in the lowest adherence quartile. Analysis of data for over 47,000 participants in the Nurses’ Health Study and the Health Professionals Follow-up Study in the USA found that adherence to a Mediterranean-style diet was associated with a delay in the onset of is some prodromal nonmotor PD symptoms (160). A large study from China including over 71,000 participants examined the association between overall diet quality and prodromal PD features (161). Diet quality was assessed using the modified Alternative Healthy Eating Index and alternate Mediterranean diet (aMED). Better diet quality was associated with lower probability of having prodromal PD features, including daytime sleepiness and constipation. A prospective observational study, Complementary and Alternative Medicine (CAM) Care in PD, included over 1,000 subjects and found that the foods associated with the reduced rate of PD progression included fresh vegetables, fresh fruit, nuts and seeds, non-fried fish, and olive oil (162), which align with the components of a Mediterranean-style diet. A recent systematic review has suggested that dietary flavonoids, which are known to be abundant in fruits and plant-based foods, may prevent or slow PD progression due to their antioxidant properties (163). In the case of Thailand, a “Thai-Medi diet” has been proposed for people with PD to capture the recognized benefits of the Mediterranean diet but adapted to include local ingredients that maintain the unique taste of Thai food that will be acceptable to Thai citizens (158).

The importance of bidirectional communication between the gut and the brain, the so called gut–brain axis, and the composition of the gut microbiome, is the subject of considerable ongoing research interest in terms of the relationship to the pathological mechanisms that underlie PD (164, 165). It is hypothesized that PD may in fact original in the gut which aligns with observations that various types of gastrointestinal dysfunction (e.g., dysphagia and constipation) are prodromal features of PD. The composition of the intestinal microbiota in people with PD is characterized by a loss of short chain fatty acid bacteria and increased lipopolysaccharide bacteria. Diet inevitably has a huge influence on the composition of the gut microbiome and data suggest that components of a Mediterranean diet, including high levels of fiber, bioflavonoids and omega-3 fatty acids, have beneficial effects on the gut microbiome composition and intestinal cell function, which in turn has positive effects on the brain, including anti-inflammatory and antioxidant effects (164, 166). Similarly, restoration of the gut microbiome using probiotics, prebiotics or other dietary supplements has also been suggested to have the potential to slow PD progression (167).

### 3.2 Move

The best studied lifestyle intervention in terms of its potential positive impact on the development of PD, and treatment of early disease, is physical exercise, particularly vigorous aerobic exercise (153, 168, 169). The mechanisms underlying the effect of exercise on PD remain to be fully elucidated, but it is known to be associated with a higher quality of life in PD patients and is thought to result in protective and stimulatory effects that lead to better functional efficiency in higher-level cognitive networks (170). Improvements in muscle strength from regular exercise also improve balance and motor function. Physical exercise is also known to be an effective strategy to strengthen motor reserve and for developing “neuronal resilience” so that individuals are better able to cope with neurodegeneration as they age (171).

One of the histopathological characteristics of PD is the presence of Lewy bodies in brain tissue comprising aggregates of α-synuclein that are thought to contribute to the progressive loss of dopaminergic neurons (172). At a cellular level, studies have shown that exercise can induce autophagy, the process that results in lysosomal degradation of unwanted products. Studies in people with PD have shown that exercise-induced autophagy can decrease the accumulation of toxic α-synuclein aggregates, thereby delaying the progression of motor symptoms of the disease (173). Other suggested mechanisms for the effects of exercise on PD pathology include the reduction of inflammation and oxidative stress and enhancement of nerve regeneration and mitochondrial function (174).

In the clinical setting, accumulated evidence suggests that moderate-to-vigorous exercise may have a protective effect against the development of PD (175, 176). A study has also shown that the amount of exercise engagement prior to a diagnosis of PD can in fact enhance motor reserve once the disease is established, meaning that patients will have less motor deficits for similar degrees of dopamine depletion (177). Several studies have evaluated the effect of exercise in slowing the progression of early PD. SPARX (Study in Parkinson Disease of Exercise) was Phase 2, multicenter randomized clinical trial of patients with *de novo* PD who were not yet taking any medication to determine the impact of high-intensity treadmill exercise for 4 days per week (178). This exercise regimen resulted in better Unified Parkinson’s Disease Rating Scale (UPDRS) motor scores compared to patients who underwent moderate or no exercise. SPARX3, a multicenter, randomized Phase 3 clinical trial, is now ongoing to determine the ability of high-intensity endurance treadmill exercise to slow the progression of PD as assessed by evaluation of the MDS-UPDRS motor scores (179). The effects of long-term exercise intervention are currently being evaluated in the CYCLE II (Cyclical Lower Extremity Exercise for Parkinson Disease II) study a 2-site, randomized controlled trial (180). Patients will be randomized to in-home aerobic exercise sessions using an exercise bike three times per week for 12 months or usual care, and the change in the MDS-UPDRS motor score over that time will be assessed and compared in the two groups. The current consensus, therefore, is that exercise should form an essential part of the initial therapeutic strategy for early PD—the concept of “exercise as medicine”—in particular aerobic exercise and resistance training (181), but that it may also have a role in both primary and secondary disease prevention (182).

### 3.3 Sleep

According to a position statement issued by the American Academy of Sleep Medicine (AASM), sleep, of good quality and an adequate quantity, is something that is essential to people’s health and wellbeing (183). Similarly, lack of sleep, or experiencing sleep disorders that disrupt the body’s normal circadian rhythm, can have a significant negative impact on both physical and mental health (184). At a cellular level, adequate sleep is important to enable the restoration, regulation and repair of key neural process and to ensure good cognitive function, and there is now accumulating evidence that poor sleep may be associated with the development and progression of some chronic diseases, such as cardiovascular disease and Alzheimer’s disease (184), so it is probable that the same could be true for PD. Similarly, disruption of sleep and circadian rhythms are known as key factors in neurodegeneration, and their occurrence during early PD stages suggests a causal role in the disease pathogenesis (185). Notably, preclinical data suggest that a contributor to the restorative effects of sleep may be its removal of potentially neurotoxic waste products that accumulate in the central nervous system while awake (186). Recognizing the important bidirectional relationship between sleep, health and disease, the AASM recommends that healthy sleep should be a key target for education and public health initiatives. Similarly, the American Heart Association includes sleep duration as a vital component of its “Life’s Essential 8” as a metric for cardiovascular health (187). A recent review of studies in people with mild cognitive impairment or Alzheimer’s disease found that sleep problems such as obstructive sleep apnea as well as sleep duration had strong pathophysiological links with the development of dementia (188). A number of small studies have also suggested a positive relationship between sleep and physical exercise, in that exercise may reduce the negative consequences of poor sleep on cognitive function, and conversely that sleep may be a mechanism by which exercise can improve cognitive function (189). Sleep problems are common nonmotor PD symptoms and can be observed in both the prodromal and early stages (190). The most common sleep disorders are insomnia, sleep disordered breathing, excessive daytime sleepiness, RBD, and sleep-related movement disorders, such as restless legs syndrome (191). We know that RBD is a strong predictor of subsequent PD (60, 62), therefore early detection of sleep problems in older people, in particular RBD, may provide an opportunity for early intervention to treat the sleep disorder itself and to prevent the development of PD. While evidence for direct preventative effects of sleep on PD are limited, it is a logical proposition that improved sleep quality will improve overall symptoms of the affected individual and potentially delay the development/progression of PD.

People who are identified by the Parkinson’s disease risk survey model as having early signs of PD will be referred to a neurologist for clinical examination and confirmation of PD through the network of National Health Security Offices and the Thai Red Cross Society. If a diagnosis of PD is confirmed, they will be referred for treatment according to their healthcare plan (12).

The model developed for the Parkinson’s disease risk survey combines multimodal features with machine learning analysis of both questionnaire responses and results from objective measurement of symptoms to provide a composite score for the risk of PD. This multimodal approach has been used previously with high accuracy for the preclinical diagnosis of PD. While the heterogeneity of datasets obtained from multiple sources challenges the classical approach to data analysis and interpretation, it also provides new opportunities for extracting valuable information using machine learning tools. The Parkinson’s Progression Markers Initiative (PPMI) database is a good example of this, being a large archive of Big Data, both structured and unstructured (192), and the significant information that can be generated using this approach. Using machine learning tools, PPMI data for 401 subjects with early PD dopaminergic imaging markers were used to classify early PD (193). The SVM classifier used in this study resulted in an accuracy of 96.40% with 97.03% sensitivity, 95.01% specificity, and 98.88% area under ROC curve. Another study, also using data from the PPMI database, constructed a diagnostic using eight different variables, namely age, gender, family history, University of Pennsylvania Smell Inventory Test score, Montreal Cognitive Assessment score, RBD Screening Questionnaire score, levels of α-synuclein in CSF, and SNCA rs356181 polymorphism (194). The model had a high accuracy for identifying early PD with an AUC of 0.93. Several reports now suggest that digital models that integrate multidimensional data, as has been done with the Parkinson’s disease risk survey model, may in fact significantly enhance the performance of PD prediction (195, 196).

Our study uses population screening with digital technology which is associated with certain challenges and limitations. With the rapid rise in the use of digital technologies in healthcare, digital health literacy, namely the ability of an individual to interact with health information on the internet or in other digital formats, is becoming increasingly important. However, digital health literacy and competence in using these tools among older people, especially in LMICs such as Thailand, is often low (197, 198), which might impact uptake of the survey. In contrast, the use of smartphones and other digital technologies among elderly people is on the rise and reported to have positive effects on their self-rated health (199), In the case of Thailand, a survey of smartphone use undertaken in 2022 found that around 77.3% of respondents aged 50 years and over used a smartphone (200), which would suggest that adoption of digital screening in Thailand is very feasible. It is also recognized that continued training of the machine learning models used for data analysis in our study will be necessary to improve the accuracy of PD diagnosis and, importantly, to avoid overdiagnosis. Further studies will be needed to determine if the model can differentiate between PD and atypical parkinsonian disorders. Currently, the model in its present iteration cannot differentiate between PD and prodromal patients, so is unclear whether prodromal patients will be classified as PD patients or as normal subjects.

While the Parkinson’s disease risk survey is an Asian initiative, data acquired from this national study will contribute to the evidence base for disease preventive strategies globally. Sharing this information and experience is an essential part of the global collaborative effort needed to meet the challenge of PD. This approach could also serve as a model for disease prevention efforts for other types of non-communicable diseases in LMICs. The healthcare system in Thailand has been ranked highly by various international agencies including the Global Health Security Index (12, 201–203) and is therefore suitably placed to take on the challenge of disease prevention in line with the WHO’s strategic objectives for overall population health, and PD in particular (17, 41, 42).

In conclusion, the Parkinson’s disease risk survey digital platform offers a robust, validated method with high accuracy for national screening of the Thai population for signs of PD. This provides a window for timely intervention to minimize the number of cases that progress to clinical PD and effective early treatment of those confirmed with early PD. Large scale implementation is possible with a strong linkage between multiple stakeholders who are involved with the care of PD. These objectives align with calls from global organizations, such as the WHO, for a greater focus on preventative strategies for PD in efforts to stem the steep rise in prevalence. If implemented successfully, the Parkinson’s disease risk survey could act as a model for screening strategies for other non-communicable diseases in Thailand, and elsewhere.

## Ethics statement

This study was approved by the Human Ethics Committee of the Faculty of Medicine, Chulalongkorn University (Institutional Review Board No. 0804/65) and was conducted according to the tenets of the Declaration of Helsinki. The participants provided their written informed consent to participate in this study. Written informed consent was obtained from the individual(s) for the publication of any identifiable images or data included in this article.

## Author contributions

RB: Conceptualization, Data curation, Formal analysis, Funding acquisition, Investigation, Methodology, Project administration, Resources, Software, Supervision, Validation, Visualization, Writing – original draft, Writing – review & editing. JS: Conceptualization, Data curation, Formal analysis, Funding acquisition, Methodology, Validation, Writing – review & editing. SP: Conceptualization, Methodology, Project administration, Writing – review & editing. CA: Data curation, Methodology, Writing – review & editing. CT: Data curation, Formal analysis, Software, Validation, Writing – review & editing. SD: Formal analysis, Software, Validation, Writing – review & editing. PP: Conceptualization, Writing – review & editing. OP: Conceptualization, Writing – review & editing. SM: Conceptualization, Writing – review & editing. VB: Conceptualization, Writing – review & editing. TP: Conceptualization, Writing – review & editing. RK: Conceptualization, Writing – review & editing. PJ: Conceptualization, Writing – original draft. AChai: Conceptualization, Formal analysis, Writing – review & editing. WJa: Conceptualization, Formal analysis, Writing – review & editing. JM: Conceptualization, Formal analysis, Writing – review & editing. AChan: Conceptualization, Formal analysis, Writing – review & editing. PR: Conceptualization, Formal analysis, Writing – review & editing. PhS: Conceptualization, Formal analysis, Writing – review & editing. WJi: Conceptualization, Formal analysis, Writing – review & editing. MC: Conceptualization, Formal analysis, Writing – review & editing. YA: Conceptualization, Formal analysis, Writing – review & editing. CS: Conceptualization, Project administration, Writing – review & editing. WS: Conceptualization, Funding acquisition, Project administration, Writing – review & editing. GB: Funding acquisition, Project administration, Writing – review & editing. AP: Conceptualization, Project administration, Writing – review & editing. PiS: Conceptualization, Funding acquisition, Writing – review & editing. JV: Conceptualization, Funding acquisition, Writing – review & editing. TB: Conceptualization, Funding acquisition, Supervision, Writing – review & editing.
