# Supplementary material for: The rise of Parkinson’s disease is a global challenge, but efforts to tackle this must begin at a national level: a protocol for national digital screening and “eat, move, sleep” lifestyle interventions to prevent or slow the rise of non-communicable diseases in Thailand
Source: Front Neurol. 2024 May 13;15:1386608. doi: 10.3389/fneur.2024.1386608 (PMC11129688; doi:10.3389/fneur.2024.1386608)
Supplement: Supplementary file 1 [file Data_Sheet_1.DOCX]

**Supplementary Material 1.** Original Thai language version of the 20-item Parkinson’s Disease Risk Survey questionnaire developed for population screening in Thailand.

**แบบคัดกรองอาการโรคพาร์กินสัน**

|  | **ใช่** | **ไม่ใช่** |
| --- | --- | --- |
| 1. ท่านเคยมีอาการสั่นที่มือ หรือขา โดยมีอาการขณะพักหรืออยู่เฉยๆ |  |  |
| 1. ท่านเขียนหนังสือช้าลง หรือ เขียนหนังสือตัวเล็กลงกว่าเดิม |  |  |
| 1. ท่านรู้สึกว่าเคลื่อนไหวช้าลงกว่าเมื่อก่อน เช่น การหวีผม แต่งตัว อาบน้ำ หรือรู้สึกว่าการ ติดกระดุม หรือเปิดฝาขวดน้ำ ทำได้ลําบากกว่าเก่า |  |  |
| 1. เคยมีคนบอกว่าเสียงของท่านเบาลงกว่าเมื่อก่อน หรือผู้ฟังต้องถามซ้ำบ่อย ๆ เพราะ ไม่ได้ยินเสียงท่านพูด |  |  |
| 1. ท่านรู้สึกว่าแขนของท่านแกว่งน้อยลงเวลาเดิน |  |  |
| 1. ท่านเดินก้าวสั้น ๆ และเดินซอยเท้าถี่ |  |  |
| 1. ท่านมีอาการก้าวขาไม่ออก หรือก้าวติด ขณะเริ่มต้นออกเดิน หรือกําลังเดิน หรือหมุนตัว |  |  |
| 1. ท่านมีปัญหาพุ่งตัวไปข้างหน้าขณะเดิน ทําให้ก้าวตามไม่ทัน หรือหยุดเดินทันทีได้ยาก |  |  |
| 1. ท่านมีอาการข้อใดข้อหนึ่งต่อไปนี้ พลิกตัวได้ลําบากเวลานอน หรือลุกจากที่นอนลําบาก หรือหลังจากนั่งลงแล้ว ท่านรู้สึกว่าลุกยาก หรือลุกลําบาก |  |  |
| 1. อาการสั่น เคลื่อนไหวช้า หรือแข็งเกร็งเริ่มที่ข้างใดข้างหนึ่งของร่างกายก่อน |  |  |
| 1. ท่านทราบว่าตนเองมีอาการพูดออกเสียง หรือตะโกน หรือละเมอ ขยับแขนขาที่อาจจะ สอดคล้องกับความฝัน หรือตกเตียงขณะนอนหลับ หรือเคยได้รับการบอกจากคู่นอน หรือผู้ดูแล ว่าท่านมีอาการดังกล่าว |  |  |
| 1. ท่านมีอาการง่วงนอนระหว่างวันมากผิดปกติ หรือผลอยหลับระหว่างขณะทํากิจกรรม เป็นประจำ |  |  |
| 1. ท่านรู้สึกว่าการได้กลิ่นของท่านลดลง |  |  |
| 1. ในช่วง 3 เดือนที่ผ่านมา ท่านมีอาการท้องผูกเรื้อรัง โดยถ่ายน้อยกว่า 3 ครั้ง/ สัปดาห์ |  |  |
| 1. ท่านมีอาการซึมเศร้า ร้องไห้ง่ายกว่าปกติ หรือขาดความสนใจต่อสิ่งแวดล้อมรอบข้าง หรือสิ่งที่เคยให้ความสนุกสนานในอดีต |  |  |
| 1. ท่านเคยเห็นภาพหลอน หรือได้ยินเสียง โดยที่ไม่มีตัวคน |  |  |
| 1. ท่านมีอาการหน้ามืด มึนหรือเวียนศีรษะ เวลาเปลี่ยนท่าจากนั่งหรือนอนเป็นลุกยืน และอาการมักจะดีขึ้นหรือหายไป หลังจากที่นั่งหรือนอน เป็นประจำ |  |  |
| 1. ท่านมีปัญหาการควบคุมปัสสาวะ เช่น กลั้นปัสสาวะไม่ได้ ปัสสาวะคั่ง เป็นประจำ |  |  |
| 1. ท่านมีปัญหาความคิดวิเคราะห์ ความจํา การคํานวณ ที่แย่ลง นานมากกว่า 1 ปีขึ้นไป |  |  |
| 1. ท่านมีปัญหาการทรงตัว หรือหกล้มบ่อย ตั้งแต่ในระยะแรกที่เกิดอาการ เคลื่อนไหวช้า แข็งเกร็ง หรือ สั่น |  |  |
